# In Situ 4D STEM of LiNiO_2_ Particles Heated in an Oxygen Atmosphere: Toward Investigation of Solid‐State Batteries Under Realistic Processing Conditions

**DOI:** 10.1002/smtd.202500357

**Published:** 2025-05-13

**Authors:** Thomas Demuth, Shamail Ahmed, Philipp Kurzhals, Johannes Haust, Jürgen Belz, Andreas Beyer, Jürgen Janek, Kerstin Volz

**Affiliations:** ^1^ Department of Physics and mar.quest (Marburg Center for Quantum Materials and Sustainable Technology) Philipps‐University Marburg 35032 Marburg Germany; ^2^ Institute of Physical Chemistry and Center for Materials Research Justus‐Liebig‐University (JLU) 35392 Giessen Germany; ^3^ BASF SE New Battery Materials and Systems 67056 Ludwigshafen am Rhein Germany

**Keywords:** 4D STEM, in situ, LiNiO_2_, rock‐salt phase, solid‐state battery

## Abstract

Cathode active material (CAM) particles and solid electrolyte (SE) – CAM composites for solid‐state batteries (SSBs) are often subjected to elevated temperatures during annealing or co‐sintering. This thermal treatment can affect the material's structure and induce degradation processes, particularly at the SE – CAM interface. To better understand these phenomena and improve material stability and performance, investigations by (scanning) transmission electron microscopy ((S)TEM) under realistic processing conditions, i.e., in an oxygen atmosphere, are desirable. However, preparing electron‐transparent TEM lamellae of SE – CAM composites with intact interfaces is highly challenging. Therefore, an in situ heating methodology is first established using LiNiO_2_ (LNO) particles as a model system. In this study, the morphological and structural evolution of thinned LNO particles during heating in an oxygen atmosphere is investigated, employing in situ 4D nanobeam STEM. The in situ observations are complemented with *postmortem* electron diffraction and spectroscopy measurements. These findings indicate that LNO undergoes structural degradation at temperatures ≈350 °C, transitioning from the layered ($R\bar{3}m$) structure to a NiO‐type rock‐salt phase ($Fm\bar{3}m$). This onset temperature is significantly higher than that observed in comparable in situ heating experiments conducted in vacuum, highlighting the importance of an oxygen atmosphere for replicating real‐world processing conditions.

## Graphical abstract

An experimental protocol, including sample preparation and experimental setup, for heating large, thinned cathode active material particles in situ in an oxygen atmosphere is presented. Using 4D scanning transmission electron microscopy nanobeam diffraction and ex situ measurements, the structural evolution of LiNiO_2_ during heating in oxygen is investigated. The oxygen atmosphere affects the onset temperature of degradation, differing from similar experiments conducted in a vacuum.

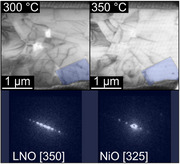

## 1 Introduction

In recent years, the shift from fossil fuels and coal to renewable energy sources, alongside the electrification of the automotive sector, has significantly increased the demand for high‐capacity energy storage solutions.^[^ ^1^, ^2^ ^]^

State‐of‐the‐art lithium‐ion batteries (LIBs) can achieve very high specific energies when utilizing layered transition‐metal (TM) oxides of the general composition LiTMO_2_ (TM = Ni, Co, Mn, Al, etc.) as cathode active materials (CAM). These materials show high specific capacities exceeding 200 mA h g^−1^, depending on the Ni content.^[^ ^3^, ^4^ ^]^ Current research focuses on further enhancing the specific capacity for high‐performance applications by further increasing the Ni content in the CAM to the extreme.^[^ ^5^ ^]^ While the use of flammable liquid electrolytes in LIBs poses a safety risk in the event of a thermal runaway,^[^ ^6^, ^7^ ^]^ the use of an inorganic solid electrolyte (SE) may mitigate this risk. Moreover, SEs enable the possibility of utilizing lithium metal as anode material, thereby significantly increasing the theoretical specific cell energy due to the high theoretical capacity of lithium metal of 3,860 mA h g^−1^.^[^ ^8^, ^9^ ^]^ For the widespread commercialization of solid‐state batteries (SSBs), several remaining challenges must be overcome.^[^ ^10^ ^]^ A key issue is the high interfacial impedance between SE and CAM, as well as between SE and anode, caused by chemical, electrochemical, and mechanical instabilities.^[^ ^11^, ^12^ ^]^ To reduce interfacial impedance, intimate contact between SE and CAM is required, which is often achieved by co‐sintering both components.^[^ ^13^, ^14^, ^15^ ^]^ During the co‐sintering process, however, secondary phase formation at the interface can occur due to enhanced cation mobility.^[^ ^16^, ^17^ ^]^ These reactions may occur at lower temperatures than the degradation temperatures of the individual components.^[^ ^18^ ^]^

Moreover, Ni‐rich CAMs suffer from rapid capacity fade due to a phase transition from the layered ($R\bar{3}m$) to the rock‐salt phase ($Fm\bar{3}m$) during cycling.^[^ ^19^ ^]^ Consequently, intensive research is focused on developing coatings to stabilize the CAM and prevent undesired reactions while maintaining good lithium‐ion conductivity.^[^ ^20^, ^21^, ^22^ ^]^ During the coating procedure, the CAM particles are mixed with coating additives and annealed at elevated temperatures.^[^ ^23^ ^]^ However, the working principle of such coating layers is not yet fully understood.^[^ ^10^, ^24^ ^]^

Furthermore, heat treatment of delithiated CAM particles may resemble the performance degradation caused by electrochemical cycling, highlighting the critical need for a comprehensive understanding of the material's behavior during heating.^[^ ^25^ ^]^

These examples highlight the importance of studying temperature‐induced structural transformations in CAMs and SE – CAM composites to improve the performance and safety of next‐gen batteries.

A powerful method to investigate nanoscale processes in real‐time is in situ (scanning) transmission electron microscopy ((S)TEM). It allows for imaging as well as diffraction and spectroscopy measurements during the heating process. Consequently, in situ TEM has recently been extensively utilized to study battery materials.^[^ ^26^, ^27^, ^28^, ^29^, ^30^ ^]^ However, despite the numerous publications on in situ TEM experiments involving batteries, in situ studies focusing on the electrochemical behavior of CAMs remain scarce.^[^ ^31^ ^]^ In many in situ TEM heating studies, CAM particles are often examined as whole particles.^[^ ^32^, ^33^, ^34^, ^35^ ^]^ However, this approach is very limited in the study of large secondary particles, where only thin regions at the edges of the particles can be structurally analyzed, as the bulk regions are too thick to transmit electrons. On the other hand, in situ TEM experiments with thinned particles are usually conducted in vacuum, which does not accurately replicate real‐world heating conditions, as co‐sintering and annealing processes are often performed in air or an oxygen‐rich environment to prevent oxygen loss from the sample.^[^ ^25^, ^36^, ^37^, ^38^, ^39^, ^40^, ^41^ ^]^ In situ TEM heating experiments of SE – CAM composites under oxygen conditions have not been – to the best of our knowledge – reported so far.

To enable such investigations under real‐world thermal processing conditions, we developed an experimental protocol to investigate solid‐state battery components in situ in the STEM during heating in an oxygen environment at atmospheric pressure. The ultimate goal of this setup is to enable direct investigation of SE – CAM interfaces during heating. However, the preparation of SE – CAM composite TEM lamellae with intact interfaces is highly challenging – even for ex situ measurements – as detailed in our previous study.^[^ ^42^ ^]^ Therefore, in this study, we focused on establishing the experimental procedure, demonstrating its feasibility, and obtaining reference data using the CAM LiNiO_2_ (LNO) with large primary particles as a model system. These reference measurements on LNO particles represent an essential intermediate step toward future studies of SE‐CAM composites under realistic thermal processing conditions. By employing a nanobeam STEM mode, we can quickly switch between STEM image acquisition and 4D scanning nanobeam diffraction (SNBD) data collection using a fast direct electron detector (DED). This enables real‐time observation of morphological changes and structural transformations within the bulk of the primary particles during heating. This approach offers the unique capability to correlate surface and bulk effects, a level of insight not achievable with other measurement techniques such as conventional in situ STEM, or in situ X‐ray diffraction (XRD). These observations are complemented with *postmortem* scanning precession electron diffraction (SPED), STEM, electron energy loss spectroscopy (EELS), and energy‐dispersive X‐ray spectroscopy (EDXS) measurements for a comprehensive analysis. As a key result, we found that the onset temperature of material degradation is significantly higher compared to similar heating experiments conducted in vacuum, illustrating the benefits of replicating real‐world heating conditions.

## 2 In Situ Setup

**Figure 1:**
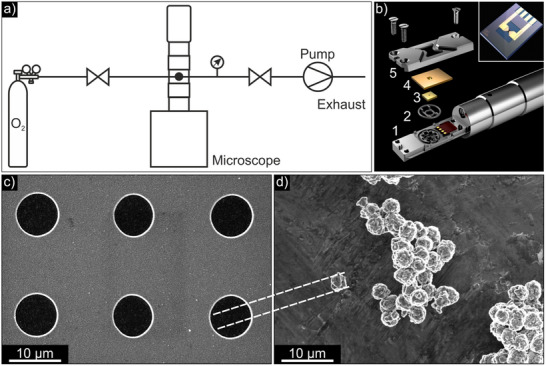
a) Schematic representation of the in situ setup. A gas bottle provides oxygen gas. The flow of oxygen is controlled by a flow valve in front of the TEM holder and the pressure in the holder is controlled by another valve downstream of the holder. A pump removes excessive oxygen. b) Rendered illustration of the Protochips Inc. Atmosphere holder tip. The inset depicts the heating MEMS e‐chip. Courtesy of Protochips Inc. c) SEM image of the Si_3_N_4_ windows of the MEMS e‐chip. d) SEM image of the investigated LNO particles.

Heating a CAM particle in an oxygen environment inside the TEM requires a dedicated experimental setup. We use a Protochips Inc. Atmosphere closed gas cell holder, which allows samples to be heated up to temperatures of 1000 °C under a gas pressure of up to 1 bar.^[^ ^43^ ^]^ To deliver oxygen to the holder, we built a custom‐made gas supply system, since the commercial setup available in our group is also used for in situ metal organic vapor phase epitaxy (MOVPE) experiments,^[^ ^44^, ^45^ ^]^ and thus contamination with toxic precursor gases had to be avoided. This new setup contains only the most important components of the commercially available setup, which are schematically represented in **Figure** **1**a, and is therefore a cost‐efficient alternative. Here, oxygen gas is provided from a gas bottle. A flow valve in front of the holder is used to digitally control the flow of oxygen into the tip of the holder to 0.1 mL min^−1^. The oxygen pressure in the lines is measured downstream of the holder, where a second valve automatically releases excessive oxygen when the set pressure of 1 bar is exceeded. The excessive oxygen is then removed by a pump. A detailed description of the components used in our setup is provided in Figure S1 (Supporting Information). Figure 1b depicts the holder tip (1). Inside the tip, a gas inlet and outlet direct the flow of oxygen into the sample volume between two micro‐electro‐mechanical systems (MEMS) chips. The sample is located on the inside of the larger heating e‐chip (4), which is also depicted in the inset of Figure 1b. It features a 120 nm thick silicon carbide (SiC) heating membrane in which six electron‐transparent 30 nm thick silicon nitride (Si_3_N_4_) windows for observation with the electron beam are embedded. A smaller window chip (3) encloses the gas cell, which is sealed by O‐ring gaskets (2) and held together by a top plate (5).

## 3 Preparation of a FIB Lamella on the MEMS E‐Chip

Due to their thickness of several micrometers and their composition of multiple agglomerated primary grains with different crystallographic orientations, the secondary LNO particles cannot be directly investigated by (S)TEM techniques by dispersing them onto the MEMS e‐chip. Instead, to enable structural investigation of a single LNO secondary particle during the heating process, the particle must first be thinned to achieve electron transparency and subsequently placed onto one of the six Si_3_N_4_ windows of the MEMS e‐chip. This approach additionally allows for site‐specific analysis of individual primary grains within the secondary particle. However, placing a thinned particle onto a Si_3_N_4_ window poses specific challenges for the sample preparation process, as the Si_3_N_4_ windows with a diameter of 9 µm are significantly larger than the LNO particles that measure less than 5 µm in diameter. This becomes apparent in the scanning electron microscope (SEM) images presented in Figure 1c,d, which were recorded at the same scale.

To securely fix the thinned particle onto a Si_3_N_4_ window while avoiding contamination or redeposition onto the particle's surface or the Si_3_N_4_ membrane during placement, and to prevent any milling‐induced damage to the window, we prepared a support frame. This frame is larger than the Si_3_N_4_ window and holds the particle in place. This strategy enables precise placement of the particle onto the window without any of the aforementioned risks. A detailed description of the sample preparation process is provided in the Supporting Information.

**Figure 2:**
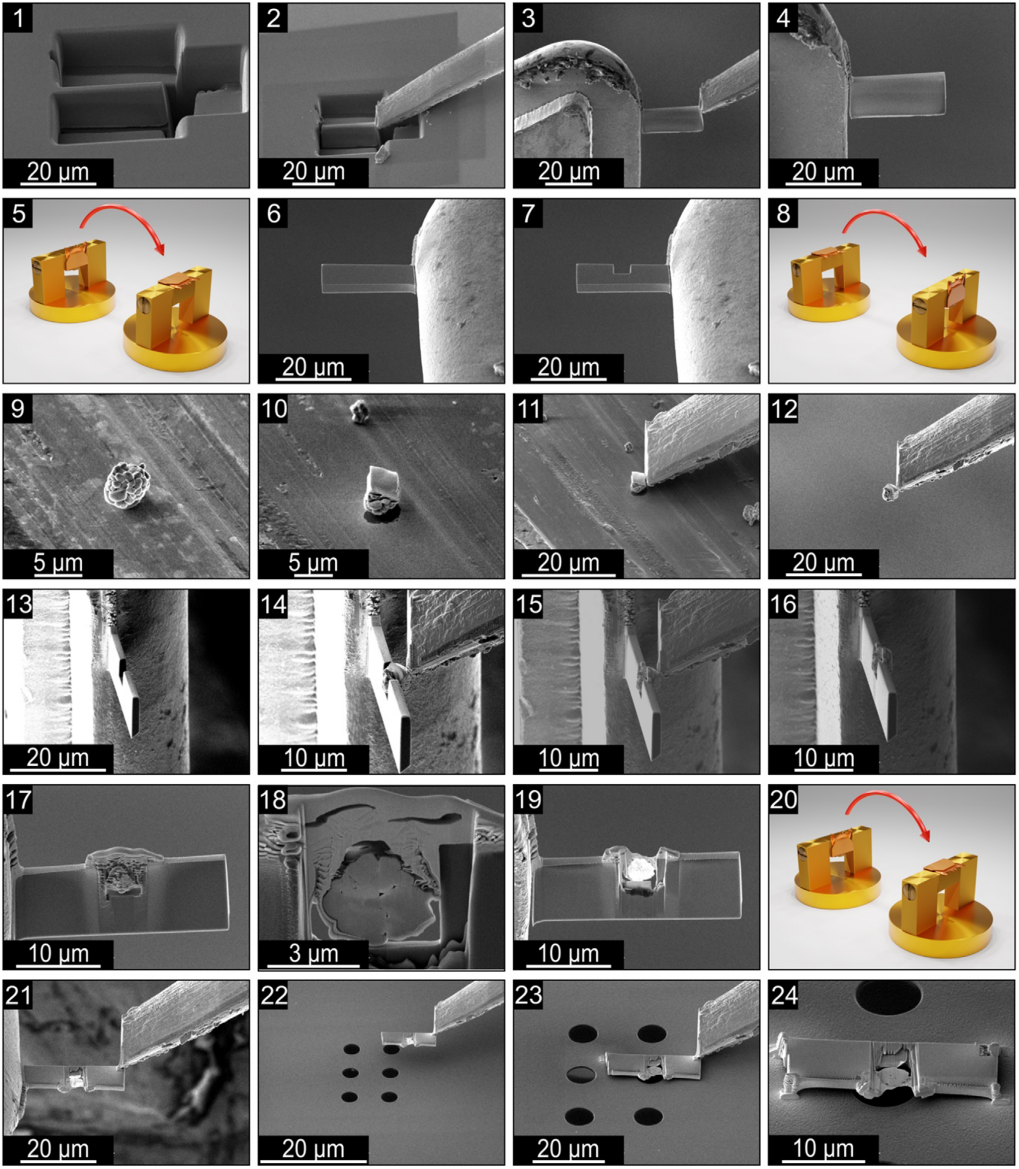
SEM images taken during the sample preparation and 3D renderings of the SEM grid holder to illustrate the orientation of the grid during preparation.

For this purpose, a lamella made of a material with good thermal conductivity and stability, as well as chemical stability toward LNO, was prepared using a standard FIB preparation routine (see **Figure** **2**(1–4), as described in detail in our previous study.^[^ ^42^ ^]^ We used silicon and gold as frame materials. However, we emphasize that other materials may also be used depending on the material under investigation.

Using a rotatable TEM grid holder, shown in Figure 2(5), a cavity was milled into the lamella (see Figure 2(6–8)), into which a single LNO particle was carefully placed using a micromanipulator needle (Figure 2(9–16)). The particle was previously covered for protection and then fixed to the frame using tungsten from the gas injection system (GIS). To achieve electron transparency of the particle without compromising the structural integrity of the supporting frame, the particle was carefully and selectively thinned while keeping the surrounding frame intact. Once sufficiently thin, the lamella was removed from the grid in a horizontal orientation using the micromanipulator needle (Figure 2(20,21)) and placed on top of an electron transparent Si_3_N_4_ window on the MEMS e‐chip, such that the LNO particle is positioned directly above the window (Figure 2(22,23)). Finally, the corners of the lamella were attached to the MEMS e‐chip using tungsten deposition from the GIS to ensure that the sample remains fixed during heating (Figure 2(24)).

## 4 Results and Discussion

**Figure 3:**
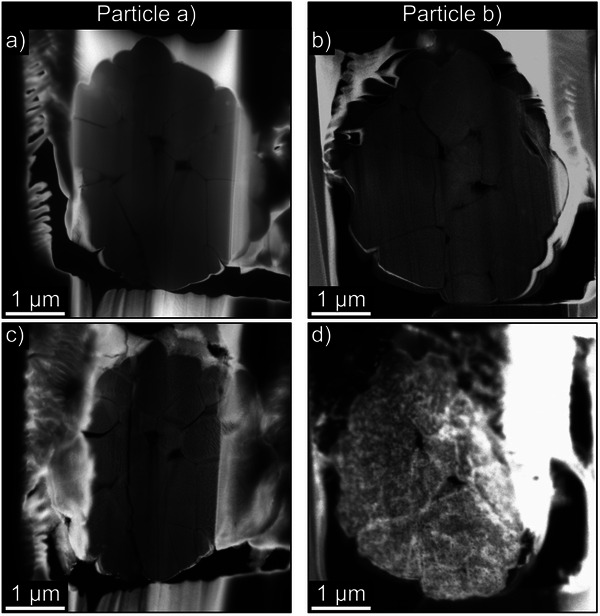
STEM high‐angle annular dark‐field (HAADF) images of the two particles before a,b) and after heating to c) 400 °C, d) 700 °C.

After successful sample preparation, the LNO secondary particle can be heated in the TEM to study the effect of the heating process in an oxygen environment on the particle structure, microstructure, and morphology. For a comprehensive analysis, we experimented with different parameters during preparation and the heating procedure. Detailed information about the process settings is provided in Table S1 (Supporting Information). The two particles that were investigated are depicted in **Figure** **3**, both before and after heating. Throughout the heating experiments, we continuously recorded high‐angle annular dark‐field (HAADF) STEM images, which were subsequently compiled into time‐lapse videos available in the supporting information. Henceforth, the particles will be referred to as Particle a) and Particle b) as defined in Figure 3.

### 4.1 Investigation of Morphological Changes by In Situ HAADF STEM

To examine the morphological changes of Particle a) during heating, we recorded HAADF STEM overview images that were compiled into Video S1 (Supporting Information). For the analysis of structural changes, we obtained 4D SNBD datasets at specific temperature intervals that are listed in Table S1 (Supporting Information). In 4D SNBD, the sample is scanned using a pseudo‐parallel beam, and a diffraction pattern is recorded at each scan point with a DED CCD camera, resulting in a 4D dataset for each temperature hold. The use of SNBD datasets is a good compromise between captured intensity and acquisition time as recording a dataset takes only a few tens of seconds. This short acquisition period, in contrast to the much longer time needed for acquiring a SPED dataset, is advantageous as the temperature of the sample is maintained only for brief intervals during SNBD data collection. Moreover, the nanobeam mode enables seamless switching between HAADF STEM imaging and SNBD dataset acquisition without requiring realignment of the beam, which further streamlines the workflow.

**Figure 4:**
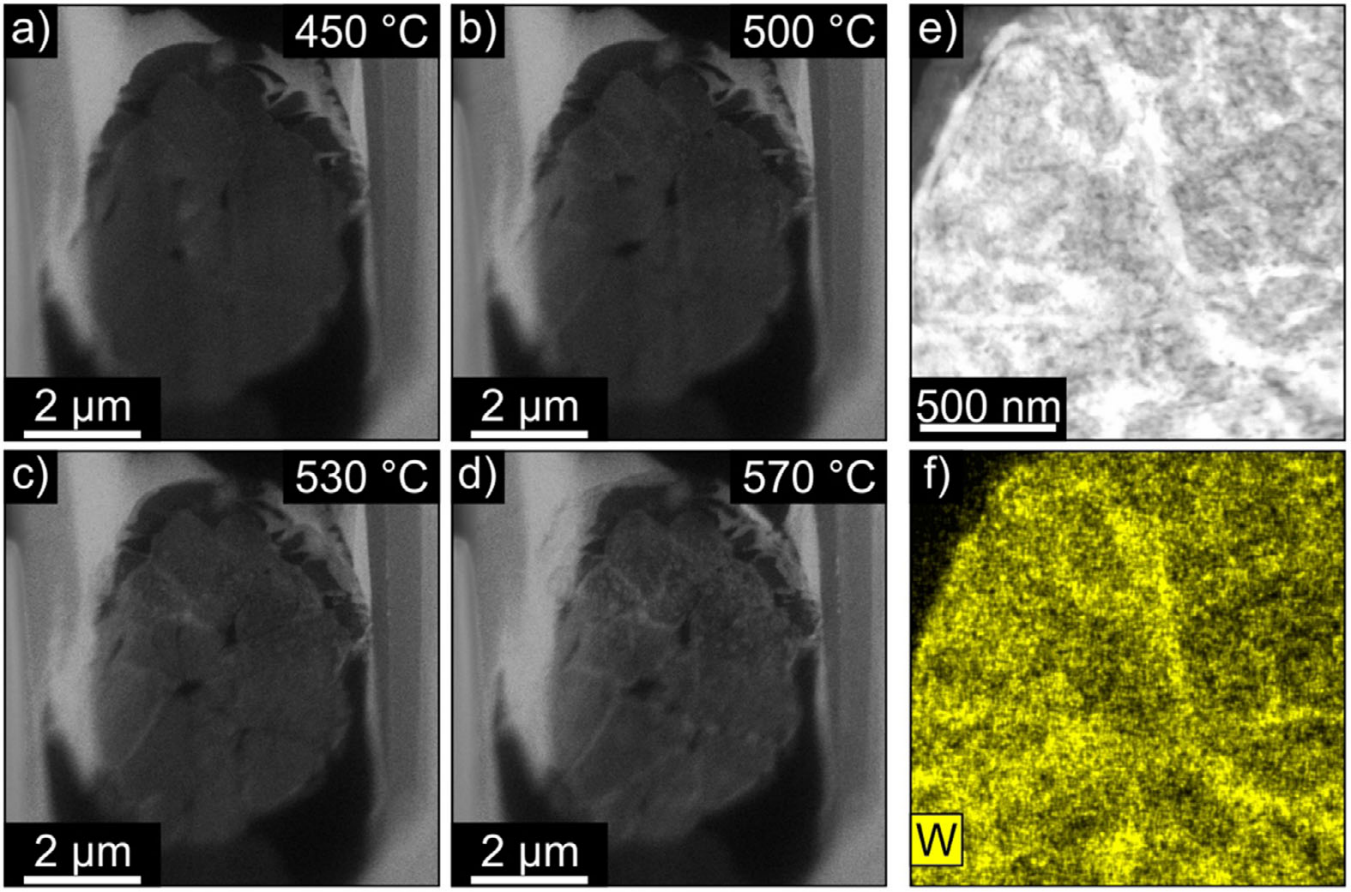
a–d) Depiction of tungsten diffusion during the heating process. e) HAADF STEM image and f) corresponding EDXS map of Particle b) heated up to700 °C.

In Video S1 (Supporting Information), noticeable changes in the particle morphology begin at the grain boundaries between primary particles ≈350 °C. A darkening near the grain boundaries causes these regions to appear broader. In some grains, this creates a shell‐like appearance. Additionally, tightly connected grains become separated. The same applies for coherent grain boundaries within primary‐like particles, as termed by Lee et al., where the true primary particles undergo splitting. These phenomena are highlighted in Figure S2 (Supporting Information).^[^ ^46^ ^]^ Above 350 °C, the surface morphology undergoes considerable changes: previously smooth surfaces become roughened, and small surface cracks emerge. These morphological changes align with observations reported by Guilmard et al. for deintercalated bulk LNO.^[^ ^47^ ^]^ At 400 °C, the particle's integrity begins to deteriorate, which led us to halt the heating process to prevent further damage to the particle. In the experiment with Particle b), the particle's condition allowed us to exceed this temperature so that we heated the particle to 700 °C. Video S2 (Supporting Information) depicts how at temperatures above 450 °C the grain boundaries at the secondary particle's surface begin to lighten up, which subsequently propagates inwards along the grain boundaries with increasing temperatures. Later, above 550 °C, the primary grains also light up. The HAADF STEM images depicted in **Figure** **4**a–d are selected frames taken from Video S2 (Supporting Information) to highlight this process. An enhanced brightness in HAADF STEM images indicates the presence of heavier elements that scatter more electrons (*Z*‐contrast).^[^ ^48^ ^]^ The EDXS map of Particle b) post‐heating, depicted in Figure 4f, validates this assessment and reveals that tungsten has accumulated on the sample, particularly along the grain boundaries. When considering only Ni, O, and W, W constitutes 24% of the material in a bulk region of a primary grain and 67% along the grain boundaries. For Particle a), which was only heated to 400 °C, only traces of tungsten (≈1%) are found. The same small amount of roughly 1% is also found on pristine, unheated LNO. In both cases, the presence of very small amounts of tungsten arises from tungsten redeposition during ion beam milling in the sample preparation process. Here, tungsten acts as a protective deposition layer and mitigating contamination of the sample during thinning is almost impossible. Therefore, the significantly higher amounts of W found on Particle b) can be attributed to tungsten diffusion, confirming our observation in Video S2 (Supporting Information) that tungsten diffusion starts only above 450 °C. That tungsten accumulates mainly along the grain boundaries agrees with previous reports in the literature investigating W‐doped Ni‐rich CAMs, which state that WO_3_ or Li*_x_* W*_y_* O*_z_* are found preferentially along the grain boundaries between primary particles.^[^ ^49^, ^50^ ^]^

Due to this unwanted interaction with the deposition layer at temperatures higher than 400 °C in the experiment with Particle b), we concentrate on the results of the experiments with Particle a) in the following.

### 4.2 Investigation of Structural Changes by In Situ Nanobeam Diffraction

**Figure 5:**
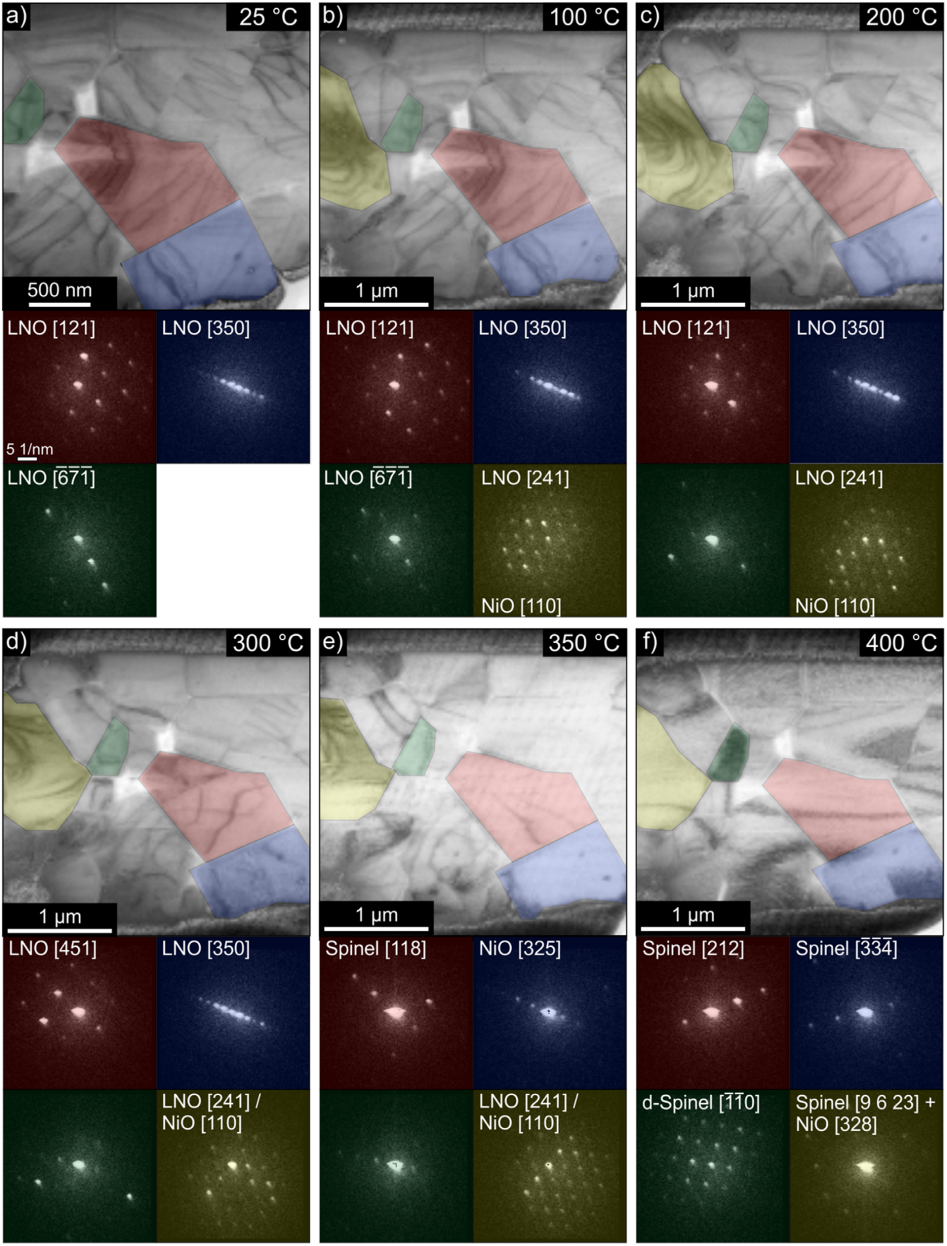
4D SNBD datasets recorded at different temperatures with corresponding diffraction patterns taken from the colored primary particles. The scale remains the same for all diffraction patterns shown. If possible, the closest approximation of the crystal structure and zone axis orientation is given according to the diffraction pattern. For the yellow‐marked particle, two possible orientations match the diffraction pattern structurally.

The morphological changes observed in Video S1 (Supporting Information) can be correlated with the structural information obtained with 4D SNBD. **Figure** **5** depicts the reconstructed virtual brightfields (VBF) of each temperature step, alongside exemplary diffraction patterns of four primary particles. The particles and diffraction patterns are color‐coded to visualize which diffraction pattern corresponds to which particle. The diffraction patterns were indexed using the software “Index 2.”^[^ ^51^ ^]^ For this purpose, simulated templates of layered LiNiO_2_ ($R\bar{3}m$), spinel Li(NiO_2_)_2_ ($Fd\bar{3}m$), distorted spinel (d‐spinel) Li(NiO_2_)_2_ (*Imma*), and NiO rock salt ($Fm\bar{3}m$) were loaded into the software and compared with the recorded diffraction patterns. The best match is shown as a label in Figure 5. Additionally, screenshots of the best‐matched phase for each orientation are presented in the Supplemental Information (Figure S3, Supporting Information). It should be emphasized that these do not represent absolute truths. Since the phase transition from layered LNO to NiO rock salt proceeds continuously, each label represents the best approximation to one of the reference phases within a spectrum of possible structural states. In cases where no label is shown, the diffraction pattern did not provide sufficient information to be confidently assigned to one of the phases.

The dark patterns observed within the primary particles in the VBFs result from subtle internal misorientations of the lattice, to which the nanobeam mode is highly sensitive. These features are also visible in the STEM images compiled into Video S1 (Supporting Information), which were recorded using the nanobeam mode. In contrast, they are not observed in conventional STEM mode, as demonstrated by the HAADF STEM image in Figure 3a. When the sample undergoes thermal expansion or bending, these patterns change in response to the external forces acting on the particles. The evaluation of the diffraction data is impeded by thermal expansion, leading to particle movement and bending. Nonetheless, several conclusions can be drawn from the data: Initially, the layered LiNiO_2_ phase is predominant, as evidenced by the indexed diffraction patterns of the colored particles. The diffraction pattern of the yellow‐marked particle cannot unambiguously be attributed to the LiNiO_2_ phase, as the [241] orientation of LNO appears to be almost identical to NiO in [110] when viewed from this direction. A representation of both structures is depicted in Figure S4a (Supporting Information). However, it is nearly impossible that the entire primary grain lacks lithium at room temperature and has transformed to NiO. Thus, the LNO [241] orientation is with certainty the correct match, although both orientations match the recorded pattern. Until 300 °C, the structure remains unaltered, with minor changes in the diffraction patterns attributed to particle movement and bending due to thermal stresses. Structural modifications begin ≈350 °C, as demonstrated by the diffraction pattern of the blue‐highlighted particle. Here, the LiNiO_2_ phase in the [350] orientation phase transitions to a NiO phase in the [325] zone axis evidenced by the disappearance of the [0 0 3*n*] (with *n* uneven) diffraction spots. This transition occurs due to Ni atoms occupying Li positions within the Li layers (see Figure S4b, Supporting Information), together with the release of oxygen. Moreover, the diffraction pattern colored red matches the spinel [118] orientation. Between 350 and 400 °C, the sample bent strongly. As the particles are now oriented differently, the depicted diffraction patterns do not resemble the diffraction patterns of lower temperatures. However, the patterns can still be attributed to degradation products. The diffraction pattern of the particle colored yellow corresponds to a superposition of spinel ($Fd\bar{3}m$) and rock‐salt ($Fm\bar{3}m$) phases. The particles colored blue and red exhibit a spinel structure, whereas for the particle colored green, the distorted spinel phase (*Imma*) provides the best match. The occurrence of the distorted spinel phase is likely caused by lattice strain induced by severe lithium and oxygen loss during heating. Nevertheless, these findings clearly indicate that a phase transition has occurred.

### 4.3 *Postmortem* Analysis

**Figure 6:**
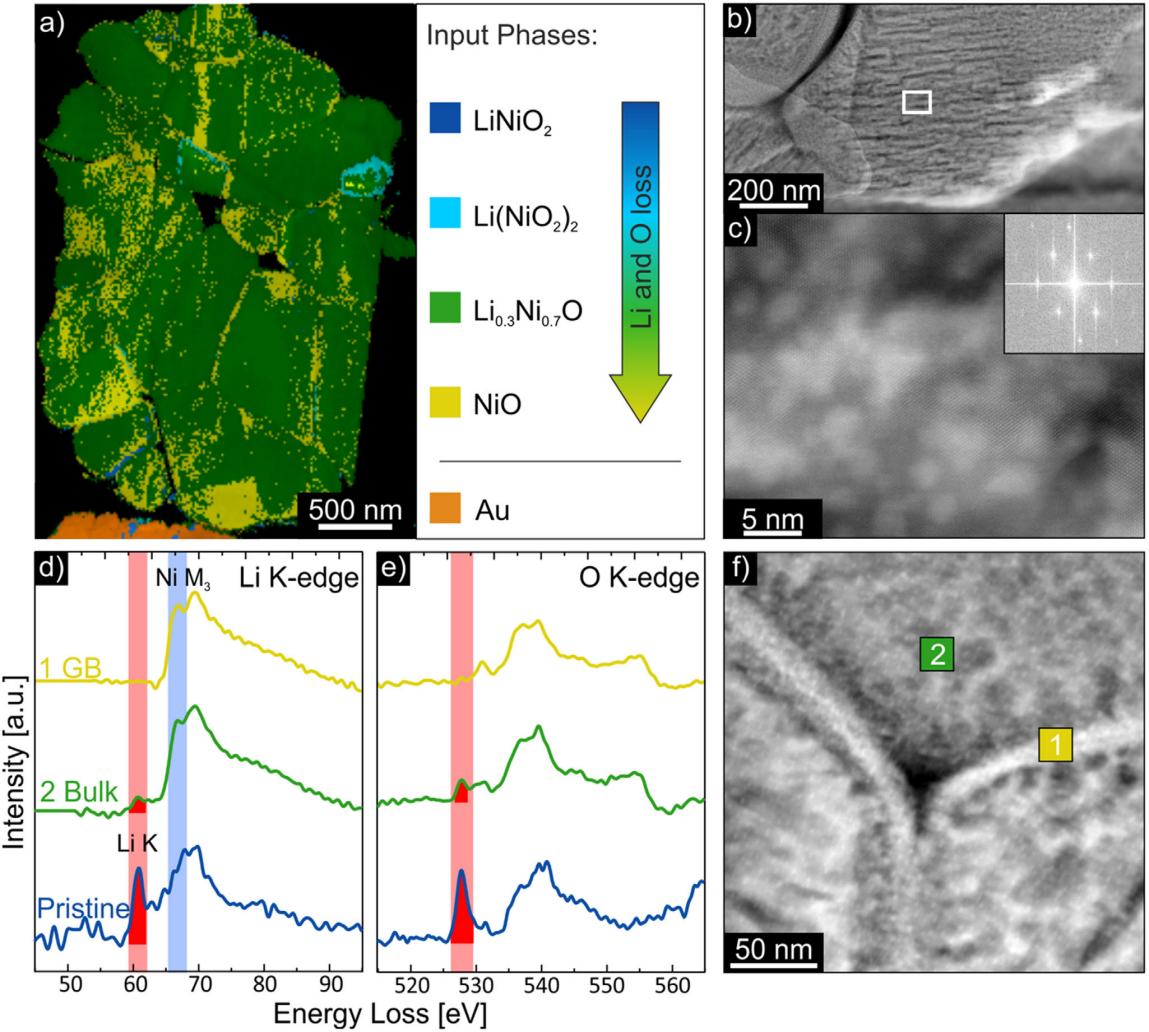
*Postmortem* results measured after the heating process. a) 4D SPED phase map of the whole LNO particle. b) STEM image showing the cracked surface of an LNO primary grain. The region marked with the white square is magnified in the HR‐STEM image in (c). In the inset, the FFT of the HR‐STEM image is given. d,e) EELS spectra of the Li K‐edge and O K‐edge recorded at the locations marked with the green squares in the STEM image shown in f) and compared with spectra of pristine LNO.

To record higher‐quality diffraction data for more reliable phase identification, we employed *postmortem* scanning precession electron diffraction (SPED). Here, the beam is slightly tilted and processed during the acquisition of diffraction patterns, which suppresses dynamical scattering effects and yields kinematical‐like diffraction patterns.^[^ ^52^ ^]^ For this purpose, we disassembled the gas‐tight holder tip and placed the heating e‐chip, on which the sample is located, into a custom‐built tip for a JEOL single‐tilt TEM holder, which has been introduced in the publication of Krug et al.^[^ ^45^ ^]^ Using this special holder tip, we avoid looking through the Si_3_N_4_ window of the window chip (labeled as 3 in Figure 1b), which is omitted in this setup, as well as the oxygen atmosphere, as the heating e‐chip with the sample is now directly placed inside the TEM column vacuum. With this setup, we recorded the 4D SPED data, which was subsequently analyzed with a template‐matching algorithm, as described by Rauch et al., which produced the phase map shown in **Figure** **6**a.^[^ ^53^ ^]^ Due to the higher quality of the diffraction patterns obtained with ex situ SPED compared to in situ SNBD, we additionally included a lithiated rock‐salt phase (Li_0.3_Ni_0.7_O) – which differs only slightly from pure NiO – in the phase evaluation besides the phases mentioned before (layered LiNiO_2_, spinel Li(NiO_2_)_2_ (both $Fd\bar{3}m$ and *Imma*), rock salt NiO). We would like to emphasize again that the results do not represent absolute phase identifications, but rather the best approximations to the input phases within a continuum of possible structures along the layered‐to‐rock‐salt phase transition. To further increase reliability, a template for gold – the material of the supporting frame – was also included in the matching process to serve as a reference for calibration. The template matching algorithm predominantly assigned rock‐salt phases, both lithiated and pure NiO, across the entire particle. While the lithiated rock‐salt phase is predominant, pure NiO can be observed at some grain boundaries around the grains. Only very few regions are identified as spinel ($Fd\bar{3}m)$ phase. The distorted spinel phase (*Imma*) was not matched (and thus not included in the phase map in Figure 6a), and only negligible traces of layered LiNiO_2_ were found.

These findings indicate that the phase transition toward NiO had progressed further in the SPED measurement compared to the in situ SNBD data, where predominantly spinel phases were identified. This can be attributed to continued heating after the SNBD dataset was recorded at 400 °C, prior to the appearance of a large crack that halted the experiment. During this time, and throughout the subsequent cooling ramp, the phase transition likely continued.^[^ ^53^, ^54^ ^]^

For additional information, ex situ HR‐STEM and spectroscopic measurements were performed. Obtaining HR‐STEM images with a single‐tilt holder is not feasible as it is quite improbable that some primary particles are oriented in such a way that they can be tilted into zone axis by just tilting one axis. We therefore decided to detach the sample with the frame from the MEMS e‐chip and attach it to a regular TEM grid that can be used with a double‐tilt holder. We held back with this step until the 4D SPED data was recorded, as we wanted to collect as much data as possible, before employing steps in which we might damage or completely lose our sample. This furthermore enabled us to collect the spectroscopy data without the influence of the Si_3_N_4_ window of the MEMS e‐chip.

We recorded HAADF HR‐STEM images of multiple grains. The LiNiO_2_ phase‐specific [nm0] orientations that show alternating Ni and Li layers (see for example Figure S4b, Supporting Information) cannot be found in any investigated grain, indicating a phase transition to the rock‐salt phase. A representative HR‐STEM image of a grain exhibiting a cubic structure is presented in Figure S5 (Supporting Information). This structure resembles the NiO phase in [100] orientation, but the LiNiO_2_ phase in [841] orientation is structurally almost identical as the inset in Figure S5 (Supporting Information) illustrates, making a definite attribution to the rock‐salt phase from the HR‐STEM image alone unfeasible. Although the HR‐STEM images we recorded may not unambiguously be assigned to the degraded rock‐salt phase, as all structures found may also be attributed to a structurally similar orientation of the layered LNO phase, the grains have surely transformed into a rock‐salt phase considering the SPED data as well. The EELS measurements discussed in the next section will give further elucidation.

The HR‐STEM images may furthermore be used to examine the morphological changes, especially the crack formation, on the primary particles’ surfaces visible in Video S1 (Supporting Information) and the VBF images in Figure 5 occurring above 350 °C. Figure 6b depicts a STEM image of a primary grain that exhibits many (close to) horizontal cracks on its surface. To compare the orientation of the cracks to the crystal structure of the primary grain we recorded an HR‐STEM image at the position of the white square in Figure 6b, which is presented in Figure 6c. The structure of the grain resembles the LNO [241] and NiO [101] orientation, which are structurally similar. This implies that the cracks in this grain occur along the {$\bar{2}10$} (LNO) or {$\bar{1}01$} (NiO) lattice planes (see Figure S6, Supporting Information). This finding is different from the observations of Wang et al., who observed crack formation in LNO to appear along the {003} planes.^[^ ^36^ ^]^ However, a difference can be expected, as the cracking along the {003} planes should take place when the sample is delithiated and simultaneously subjected to various stresses, which is not the case here.

In the HR‐STEM image shown in Figure 6c, white spots are visible on top of the grain's surface. Focusing on these spots, as shown in the HR‐STEM image in Figure S7a (Supporting Information), reveals that these spots are crystalline nanoparticles. Further EDXS investigation of a grain's surface decorated with these nanoparticles discloses that the nanoparticles consist of gold (see Figure S7b, Supporting Information). Most likely, these Au nanoparticles stem from re‐deposition of the gold frame during sample preparation. In the experiment with Particle b), we used a Si frame and did not observe nanoparticles on the surface. While both particles underwent different sample preparations (Xe‐FIB versus Ga‐FIB) and were heated to different temperatures, so that they cannot directly be compared, this finding still indicates that a frame made of Si might be better suited than Au for the preparation of the frame.

Spectroscopic measurement methods like EELS provide another means of acquiring information about the present phases. LiNiO_2_ exhibits a distinct Li peak whereas the rock‐salt phase shows little to no Li peak, depending on the residual Li content within the rock‐salt structure.^[^ ^54^, ^55^ ^]^ Moreover, the oxygen peak shape varies depending on the bonding environment. In the layered LiNiO_2_ phase, a characteristic oxygen pre‐peak at 527 eV emerges, typical of transition metal oxides.^[^ ^56^ ^]^ This pre‐peak arises from electron excitation from the O 1s state to unoccupied O 2p states, which hybridize with Ni 3d states.^[^ ^57^ ^]^ Conversely, NiO only exhibits the oxygen main peak at 538 eV, as electrons are excited from the O 1s state to hybridized O 2p and Ni 4sp states.^[^ ^35^ ^]^ In Figure 6d,e Li K‐edge and O K‐edge EELS spectra recorded at the positions marked in Figure 6f are presented and compared with the spectrum recorded from a pristine LNO particle. For better comparison of the Li K‐edge between spectra, the spectra were normalized to the Ni M_3_‐edge at 68 eV (highlighted in blue in Figure 6d). For the pristine LNO particle, the Li peak at 61 eV, as well as the O pre‐peak at 527 eV, are distinctly visible. The peak positions are highlighted with a red background and for clarity, the area under the discussed peaks is highlighted in red. For pristine LNO, the ratio of the Li K‐edge to the Ni M_3_‐edge is ≈0.8. While both the Li peak and the O pre‐peak have completely disappeared in the spectra recorded at the grain boundary of a primary particle (Li K‐edge/Ni M_3_‐edge ratio = 0), indicating that the structure has completely transformed into pure NiO rock salt, both peaks are still slightly visible in the spectra recorded in the bulk of the grain. Here, the Li K‐edge to Ni M_3_‐edge ratio is ≈0.1, which is indicative for a slightly lithiated rock‐salt phase, as described by Park et al.^[^ ^58^ ^]^ The associated uncertainties of the Li K‐edge/Ni M_3_‐edge ratios are smaller than 0.1 and do not affect the reported values when rounded to one decimal place. This observation combined with SPED and STEM results leads to the conclusion that LiNiO_2_ has transformed into NiO at the grain boundaries and Li_x_Ni_1‐x_O (x < 0.3) rock salt in the bulk regions of the grain. This is in good agreement with SPED data, where some grain boundaries are matched with the pure NiO rock‐salt phase, whereas the bulk of the grains is predominantly matched with a lithiated rock‐salt phase.

### 4.4 Discussion

All these findings underscore the occurrence and onset of profound structural and morphological changes at temperatures ≈350 °C. Once the onset temperature is reached, oxygen and lithium are released from the surface with subsequent Ni reduction, leading to a phase transformation from layered LiNiO_2_ ($R\bar{3}m$), over the spinel Li(NiO_2_)_2_ phase ($Fd\bar{3}m\;\mathrm{and}\;Imma$), to the rock‐salt phase ($Fm\bar{3}m$) due to cation disordering between TM octahedral 3a sites and now free lithium sites (octahedral 3b sites), which become occupied by Ni^2+^ ions.^[^ ^19^, ^59^ ^]^ As we do not find a lithium peak in our EELS data at the grain boundaries, it appears the expelled lithium is no longer present in the form of oxides or carbonates on the surface of the particle. Instead, it is either removed by the oxygen flow or diffuses to different regions, such as the frame holding the particle or the SiC membrane of the MEMS e‐chip, which are not electron‐transparent and therefore cannot be investigated. The loss of oxygen and lithium from the surface is accompanied by the formation of microcracks, which in turn leads to the formation of new free surfaces and the subsequent release of oxygen and lithium from deeper regions of the sample, culminating in the transformation toward the rock‐salt phase deeper in the bulk of the material.^[^ ^60^, ^61^ ^]^ This explanation aligns closely with our observations, as we identify a pure NiO rock‐salt phase at the grain boundaries between primary particles and a lithiated rock‐salt phase in the bulk region of the primary grains. Probably, the surface areas have completely transitioned into the NiO rock‐salt structure, as indicated by our HR‐STEM investigations, while in deeper regions of the particles, areas with higher lithium content are still present, giving rise to the O K‐edge pre‐peak in the EELS spectrum, as well as Li_0.3_Ni_0.7_O assigned in SPED template matching.^[^ ^34^ ^]^

The onset temperature observed at 350 °C in our study is significantly higher than those reported by other researchers who conducted in situ TEM heating experiments of LNO and Li(Ni_0.85_Co_0.10_Mn_0.05_)O_2_ in vacuum, with onset temperatures of 200 °C and 175 °C, respectively.^[^ ^36^, ^37^ ^]^ This finding is in line with the results of Karki et al., who reported increased thermal stability of Li(Ni_0.8_Co_0.15_Al_0.05_)O_2_ (NCA) in an environmental TEM operated under an oxygen overpressure of 400 mTorr compared to an oxygen‐poor or reducing environment with an onset temperature of 350 °C.^[^ ^35^ ^]^ However, it is important to note that the Co and Al substitution in NCA is known to enhance thermal stability compared to pure LNO.^[^ ^3^, ^62^ ^]^ Therefore, the observation of an identical onset temperature of 350 °C for LNO, despite the absence of stabilizing dopants, underscores the substantial stabilizing effect of the higher oxygen pressure of 1 bar in our setup. This observation is consistent with thermodynamic and kinetic considerations. The release of oxygen from the surface depends on the difference in oxygen chemical potential (Δ*µ*O) between the bulk equilibrium phase ($\mu_{s}^{O}$) and the gas‐phase equilibrium ($\mu_{g}^{O}$). According to:

(1) $$\Delta \mu^{O}=\left(\mu_{s}^{O}-\mu_{g}^{O}\right)=-\frac{1}{2}k_{B}T\;\ln\left(\frac{P_{O_{2}}}{P_{O_{2}}^{e}}\right)$$

where *k_B_* is the Boltzmann constant, Δ$\mu^{O}$ depends on the temperature *T*, as well as on the oxygen pressure in the gas phase $P_{O_{2}}$ and the equilibrium oxygen pressure in LNO $P_{O_{2}}^{e}$.^[^ ^35^, ^63^, ^64^ ^]^

Equation (1) indicates that a higher oxygen partial pressure $P_{O_{2}}$ suppresses oxygen release from the lattice, while increasing temperature promotes it. Thus, a higher oxygen partial pressure shifts the onset of oxygen loss to higher temperatures. A reduced loss of oxygen also implies that less Ni^3+^ is reduced to Ni^2+^. Since Ni^2+^ and Li^+^ have similar ionic radii and are prone to cation mixing, and because Ni^3+^ forms stronger Ni‐O bonds than Ni^2+^, a higher Ni^3+^ content in the lattice leads to a more ordered and stable structure.^[^ ^65^, ^66^, ^67^ ^]^ In combination with fewer oxygen vacancies, this reduces surface reactivity.^[^ ^65^ ^]^ These effects are enabled by our dedicated in situ TEM heating holder operating at atmospheric oxygen pressure, which better mimics realistic thermal processing conditions such as annealing or co‐sintering compared to environmental TEM holders with lower oxygen pressure.

It is worth noting that XRD studies have reported LNO bulk particle stability up to temperatures above 600 °C.^[^ ^40^, ^68^ ^]^ This discrepancy may, in part, arise from the altered geometry of our thinned lamellae. In contrast to XRD measurements performed on uncut, spherical particles, our FIB‐prepared samples exhibit a significantly higher surface‐to‐bulk ratio. Nevertheless, even in spherical particles, certain surfaces and grain boundaries are exposed to oxygen during heating, although to a lesser extent. The degradation processes occurring at these features are qualitatively the same as those in thinned samples. Yet, surface effects are naturally more pronounced in thin lamellae. For example, in a 100 nm thin lamella, a surface modification layer of 20 nm on each side already accounts for almost half of the total volume, whereas in micrometer‐sized spherical particle this would represent only a minor fraction of the total volume. Therefore, small phase transitions at the surface are more easily detected by (S)TEM, whereas XRD primarily probes the particle bulk and is largely insensitive to such surface effects. Thus, although the geometry of our sample differs from that in bulk XRD studies, it is likely that small phase transitions at the particle surface occur at lower temperatures but remain undetected in XRD until larger fractions of material have transformed.

In contrast, our in situ approach enables simultaneous observation of transformations near grain boundaries as well as within the bulk of the particle at nanometer resolution, allowing us to capture early onset changes that would otherwise remain inaccessible. Although the bulk region is naturally reduced in thinned particles, the observed differences in lithium content between grain boundary areas and the remaining bulk still allow a clear distinction between these regions. This comprehensive perspective at the nanoscale provides valuable insights into degradation mechanisms beyond merely determining onset temperatures, by directly visualizing where degradation initiates and how it propagates at the nanoscale. These insights are not accessible with other techniques such as XRD. This may help to optimize thermal processing parameters – such as annealing temperatures for coatings or co‐sintering steps – for improved material performance. Importantly, the developed experimental setup offers a basis for future in situ investigations of other solid‐state battery components under realistic processing conditions.

## 5 Conclusion

We established an experimental approach enabling the real‐time investigation of morphological and structural changes in LNO CAM particles during in situ heating in an oxygen atmosphere using STEM and a 4D nanobeam diffraction mode.

Our findings reveal that oxygen loss from the LNO sets in at temperatures ≈350 °C, resulting in the degradation of its layered structure into a rock‐salt‐type phase. This onset temperature is significantly higher than that observed in similar in situ TEM experiments conducted in vacuum, underscoring the importance of an oxygen atmosphere for mimicking real‐world annealing or co‐sintering conditions. At the same time, it is lower than the temperatures reported in XRD experiments, demonstrating the sensitivity of our method to nanometer‐scale phase transitions.

Our approach provides a powerful platform for studying the structural evolution of cathode materials and CAM‐SE composites under realistic processing conditions relevant for SSBs.

## 6 Experimental Section

### Synthesis of Secondary LNO Particles

The sample was calcined through a solid‐state synthesis route starting from the base materials Ni(OH)_2_ and LiOH·H_2_O. A commercial batch‐type Ni(OH)_2_ precursor (Hunan Zoomwe Zhengyuan Advanced Material Trade Co., Ltd.) with a secondary particle structure and *d*_50_ value of 4 µm was utilized. LiOH·H_2_O was used as a Li source (Albemarle Corporation), which was ground before the synthesis with an air classifying mill to obtain particles of ≈10–20 µm. 30 g of Ni(OH)_2_ and the respective amount of LiOH·H_2_O to get the defined number of Li equivalents per mol of Ni of 1.02 were mixed using a laboratory blender (Kinematica AG). Afterward, this premix was filled into a ceramic crucible and fired in a laboratory box‐type furnace (Linn High Therm GmbH). First, the temperature was ramped up to 400 °C and fixed for 4 h, and then the temperature was ramped up to the maximum calcination temperature *T*_max_ = 800 °C and was kept for 1 h. For both steps, a heating rate of 3 °C min^−1^ was chosen. All experiments were run in a pure oxygen atmosphere (flow rate of 100 L per hour corresponding to about ten furnace‐volume exchanges per hour). After the synthesis, the samples were cooled down to 120 °C and brought to a dry room (21 °C, dew point < −40 °C) inside a gas‐tight box to prevent reactions with ambient moisture and CO_2_. Handling of dry CAM powders was generally done in the dry room. Prior to the characterization of the materials, the powder was sieved using a sieve with a mesh size of 32 µm (Retsch GmbH).

### Sample Preparation by FIB

Particle a) was prepared using a HELIOS 5 Hydra CX. For preparation of the Au frame and placing the particle within, as well as initial thinning of the particle down to 1 µm, a Xenon beam at 30 kV (12 kV for deposition) was employed. Subsequently, thinning was continued with a 12 kV Ar beam until reaching a thickness of ≈150 nm, followed by polishing with a 5 kV Ar beam.

Particle b) was prepared with a JEOL JIB 4601F dual beam system equipped with a Ga‐ion beam. Up to a thickness of 300 nm a 30 kV ion beam was employed for thinning. Between 300 and 100 nm the beam energy was reduced to 15 kV and below 100 nm the sample was polished using a 5 kV beam.

Comprehensive details of the preparation steps were outlined in the Supporting Information.

### Investigation by (S)TEM

The in situ heating experiments were performed using a Protochips Inc. Atmosphere holder equipped with a custom‐made oxygen supply system. The MEMS e‐chips were thermally calibrated by the manufacturer (Protochips Inc.). The accuracy of this calibration has been verified in previous in situ experiments conducted by our group.^[^ ^69^ ^]^ While exact temperatures at the specimen location cannot be confirmed down to a single degree, the set temperature was considered a reliable approximation. Minor temperature gradients on the scale of a few degrees across distances of several hundred nanometers cannot be excluded. However, no indications of such gradients were observed during the experiments.

The microscope in use for the heating experiments, as well as the *postmortem* STEM, EELS, and EDXS measurements, was a double Cs corrected JEOL JEM 2200FS with an in‐column omega filter operated at an acceleration voltage of 200 kV. In STEM mode the convergence angle of the beam was 15.07 mrad and the HAADF STEM images were recorded by an annular detector in the angular range between 70 and 280 mrad.

The 4D SNBD data was recorded at each temperature hold with a DED PN CCD camera. The dwell time per pixel was set to 0.5 ms. The SNBD mode was realized by setting up the semi‐convergence angle to 1.8 mrad. The 4D data was converted into.bloc files, which were compatible with the ASTAR software package. The.bloc files were then fed into the “Index 2” software, which matches the recorded diffraction patterns to calculated diffraction patterns from bank files created with the software “DiffGen 2.”^[^ ^51^, ^70^ ^]^ Crystallographic (.cif) files for LiNiO_2_, Li(NiO_2_)_2_ ($Fd\bar{3}m$) (data retrieved from the Materials Project for Li(NiO_2_)_2_ (mp‐25388) from database version v2025.02.12.post1.), distorted spinel Li(NiO_2_)_2_ (*Imma*), and NiO were used to to create the bank files taking thermal expansion of the lattice into account for the measurements at different temperatures.^[^ ^68^, ^71^, ^72^, ^73^ ^]^

The EELS spectra were recorded with a Gatan Ultrascan camera and evaluated (correct zero‐loss centering, background correction, removal of plural scattering) using the software Gatan Digital Micrograph.

The EDXS maps were recorded using a Bruker Nano XFlash Detector 5060 and evaluated using the Esprit 2.3 software.

The 4D SPED data was recorded at a JEOL JEM‐3010 TEM operated at 300 kV acceleration voltage equipped with a TVIPS TemCam XF416FS camera. The precession and scanning of the beam were realized by the NanoMEGAS P2000 system. The recorded video was converted into rectangular 4D datasets and further into.bloc files by employing a self‐written Python code. After template matching with the “Index 2” software using templates for LiNiO_2_, Li(NiO_2_)_2_ (both $Fd\bar{3}m$ and *Imma*), Li_0.3_Ni_0.7_O, NiO, and Au,^[^ ^68^, ^71^, ^72^, ^74^, ^75^ ^]^ “MapViewer 2” was employed to analyze the automated matching and to create the phase map.^[^ ^51^, ^76^ ^]^

## Conflict of Interest

The authors declare no conflict of interest.

## Supporting information



Supporting Information

Supplemental Video 1

Supplemental Video 2

## Data Availability

The data that support the findings of this study are available from the corresponding author upon reasonable request.
