# Supplementary material for: In Situ 4D STEM of LiNiO2 Particles Heated in an Oxygen Atmosphere: Toward Investigation of Solid‐State Batteries Under Realistic Processing Conditions
Source: Small Methods. 2025 May 13;10(2):2500357. doi: 10.1002/smtd.202500357 (PMC12825361; doi:10.1002/smtd.202500357)

Supporting Information

*In Situ* 4D STEM of LiNiO_2_ Particles Heated in an Oxygen Atmosphere – Toward Investigation of Solid-State Batteries Under Realistic Processing Conditions

Thomas Demuth, Shamail Ahmed, Philipp Kurzhals, Johannes Haust, Jürgen Belz, Andreas Beyer, Jürgen Janek, Kerstin Volz*

Detailed description of the sample preparation procedure:

We prepared samples with both a JEOL JIB 4601F dual beam system as well as a HELIOS 5 Hydra CX. While the JEOL’s focused ion beam (FIB) system is equipped with a Ga-ion beam, the Helios Hydra features multiple plasma sources of which we used Xe and Ar for the preparation. As both systems produce high-quality specimens, we will explain the preparation process only based on the JEOL JIB 4601F. We will, however, refrain from listing the beam energies used during the preparation process as we believe that our beam settings may very well be adapted to other setups achieving the same final result. Here. the focus of this description lies in the elaborate sample preparation steps.

Preparing the Frame

The sample preparation starts with the preparation of the frame, in which the LNO particle will later be embedded. The size requirements for the frame are as follows. On the one hand, the frame has to be wider than the Si_3_N_4_ window so that it can be attached to the MEMS chip on the SiC region next to the Si_3_N_4_ windows. On the other hand, it has to be thinner than 3 µm to fit in the small volume between the two MEMS chips of the gas cell holder. Therefore, we prepared a lamella out of a Si wafer with the dimensions 25 µm x 10 µm x 2.5 µm. **Figure 2** illustrates the preparation process including milling trenches and an undercut using the ion beam of the FIB (1), as well as picking up the lamella with a micromanipulator needle and attaching it to the TEM grid using the gas injection system (GIS) of the FIB (2 and 3). A more detailed description of the preparation of a conventional lamella can be found in our previous work.^[42]^

The finalized Si lamella (4) is then rotated by 90° (5) so that its side can be oriented perpendicular to the ion beam (6). Depending on the FIB system, this is achieved by rotating the holder (as shown in **Figure 3**) or using a different holder in which the FIB grid is placed in a different orientation. In this position, a 4.5 µm by 4.5 µm large cavity, in which the LNO particle will later be placed, is milled using the ion beam. After this step, the frame is finished and can be rotated back to an upright orientation (8).

Placing a Particle in the Frame

After finishing the frame, an LNO secondary particle is prepared for the measurement. For this, LNO powder is sprinkled on a FIB stub. We experimented with sprinkling the LNO powder on carbon and copper tape but had problems lifting the particles later, so we just loosely sprinkled the powder on the stub. For the preparation, an isolated LNO secondary particle that is not aggregated with others is chosen. Once an isolated particle is found (9), a tungsten deposition layer for protection during thinning is applied with the GIS using first the electron beam for deposition up to a thickness of 200 nm and later the ion beam up to a thickness of 2 µm (10). Next, the secondary particle is picked up with a micromanipulator needle to be placed inside the cavity of the frame (11,12). For the insertion of the LNO particle, the TEM grid with the frame is oriented perpendicular to its regular position during the preparation of a lamella. This ensures that the frame itself does not block the path to the cavity for the needle. Furthermore, it is rotated by a couple of degrees from the perpendicular position so that the cavity can be observed in the electron beam image during particle insertion (13). Then, the LNO particle on the micromanipulator needle is carefully placed inside the cavity (14) and attached to it using the tungsten deposition from the GIS (15). When the particle is fixed to the frame, the needle is cut off (16).

Thinning of the Particle

Before thinning the particle, more tungsten is deposited to fill the cavity around the frame as much as possible with tungsten to ensure that the particle is tightly fixed (17). The challenge during thinning is to only thin the particle but not the frame underneath. Hence, only the area of the particle is exposed to the ion beam and the milling process is closely monitored to stop it before the frame is thinned (18). Then, consecutively smaller beams with lower energy are used until the particle is around 100 nm thick (19). Finally, the finished lamella is once again rotated to be in the correct orientation to be placed on the MEMS chip (20).

Placing the Particle on the MEMS Chip

The lying lamella is picked up with the micromanipulator needle (21). While the lamella is attached to the micromanipulator needle, the FIB stub with the TEM grid is replaced by a special new stub that functions like a vise and holds the MEMS chip. Afterward, the lamella is carefully lowered on top of one of the Si_3_N_4_ windows of the MEMS chips (22). When the lamella is located directly above the window, the GIS is used to fix the lamella to the chip using tungsten deposition (23). After securing the lamella on all four corners the needle is cut off. The finished result is depicted in **Figure 2** (24).

**Table S1:** Details of the experimental parameters used during the heating experiments. The letters a) and b) refer to the labels in Figure 3.

|  | Particle a) | | Particle b) | |
| --- | --- | --- | --- | --- |
| Frame Material | Gold | | Silicon | |
| Temperature range [°C] | 25 - 400 | | 25 - 700 | |
| Temperature holds [°C] | 100, 200, 300, 350, 400 | | None | |
| Heating ramp | 25 °C - 200 °C | 0.5 °C s^-1^ | 25 °C - 700 °C | 1 °C s^-1^ |
|  | 200 °C - 300 °C | 0.3 °C s^-1^ | 700 °C - 25 °C | 1 °C s^-1^ |
|  | 300 °C - 400 °C | 0.1 °C s^-1^ |  | |
|  | 400 °C - 25 °C | 0.5 °C s^-1^ |  |  |

**Figure S1:** Implementation of the *in situ* setup. Oxygen is supplied by a conventional gas bottle. A Bronkhorst EL-FLOW Prestige mass flow meter, connected via Bus/RS232 to a measurement PC running the software FlowDDE, FlowPlot, and FlowView, is used to regulate and monitor the oxygen flow. The pressure in the Protochips Atmosphere holder is controlled via a Bronkhorst HIGH-TECH E-5716-AAA and a Bronkhorst AIX-001P mass flow controller. An Edwards PM13222-820 membrane pump is used to pump off excessive oxygen.

**
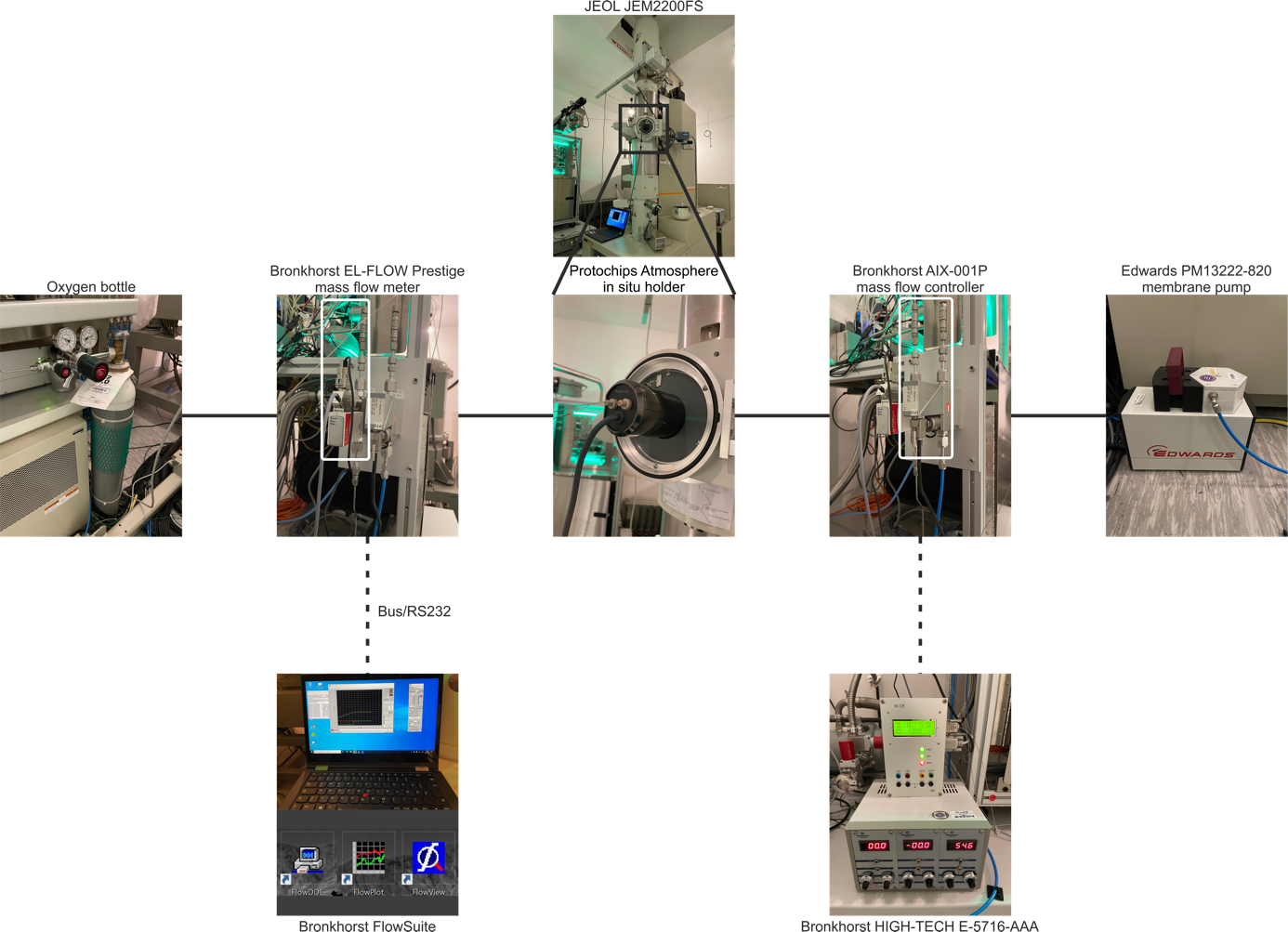
**

**Figure S2:** Examples of grain boundary evolution. Grain boundary broadening is highlighted with black arrows, grain separation of previously tightly connected grains (in part with coherent grain boundaries) highlighted with white arrows.


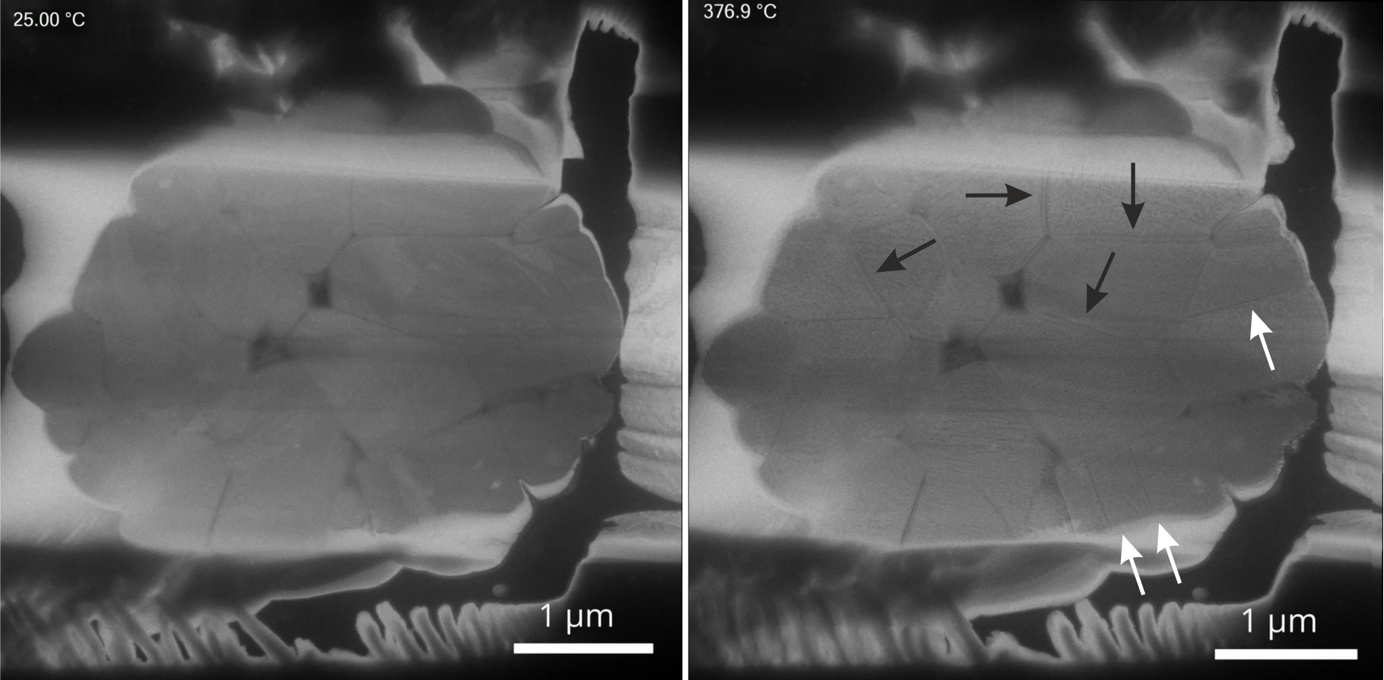


**Figure S3:** Screenshots of the Software Index (behind-window on the right) and DiffGen (front-window on the left) used for indexing the diffraction patterns shown in Figure 5. For each orientation shown in Figure 5, one example of template matching is given.


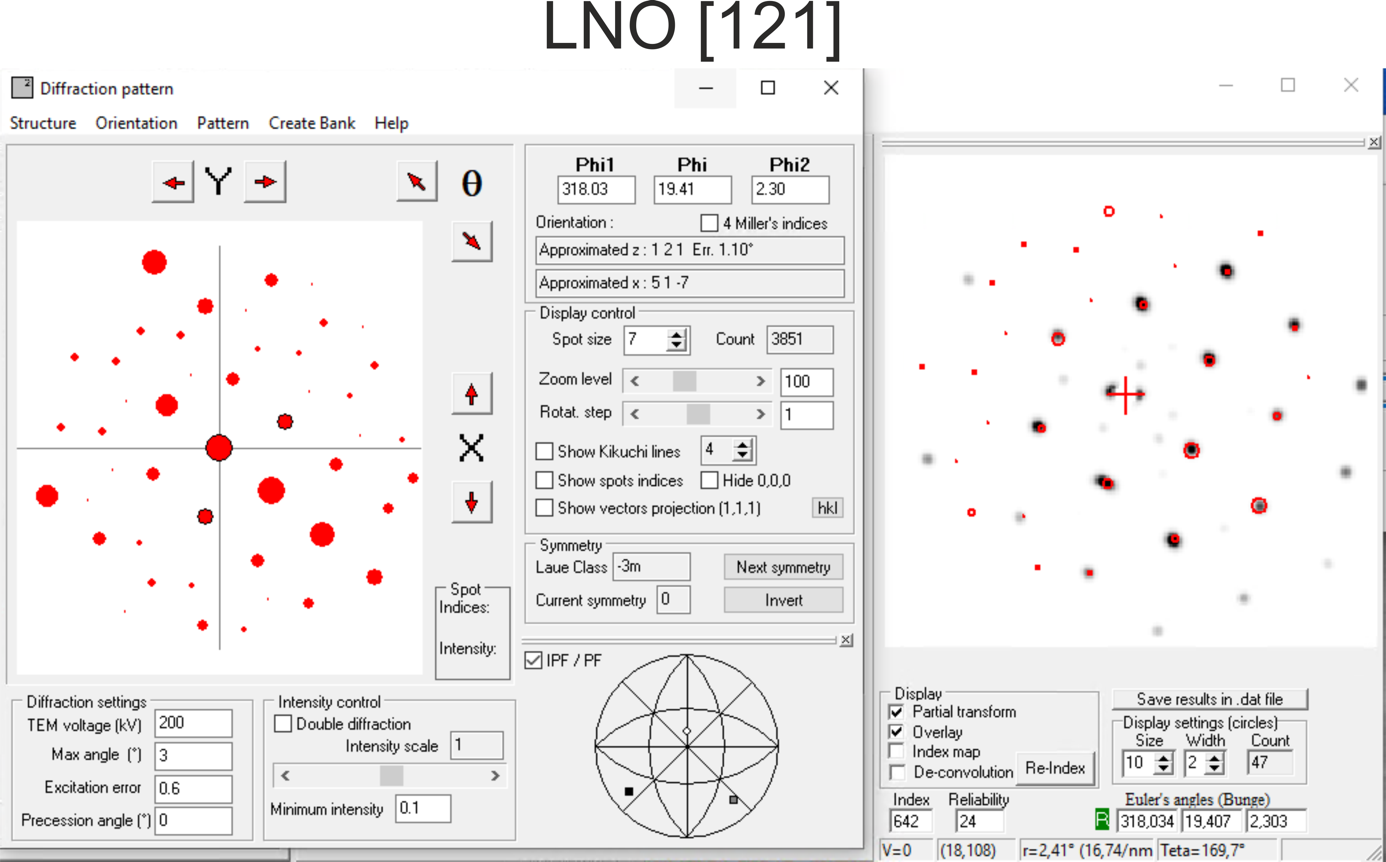


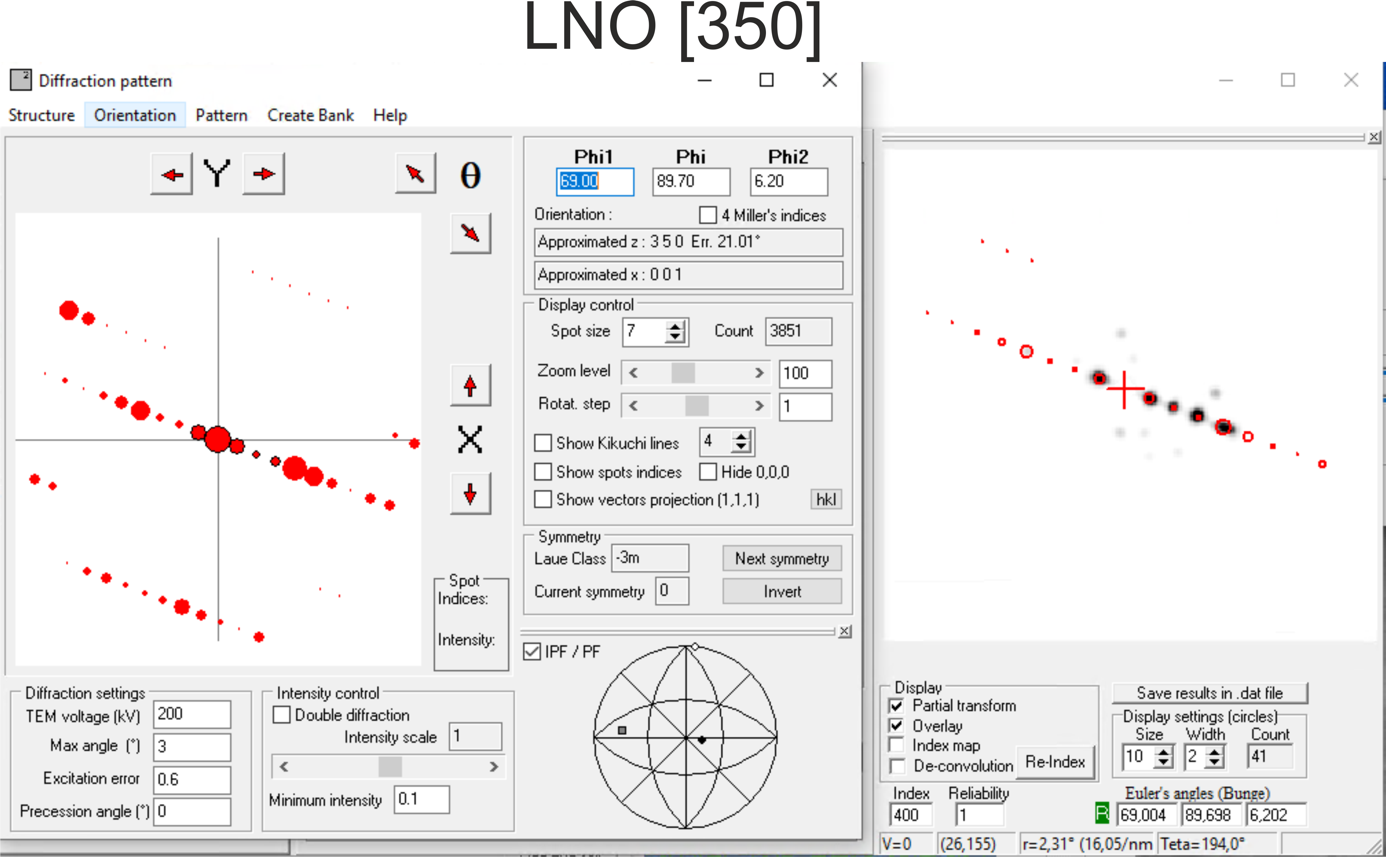


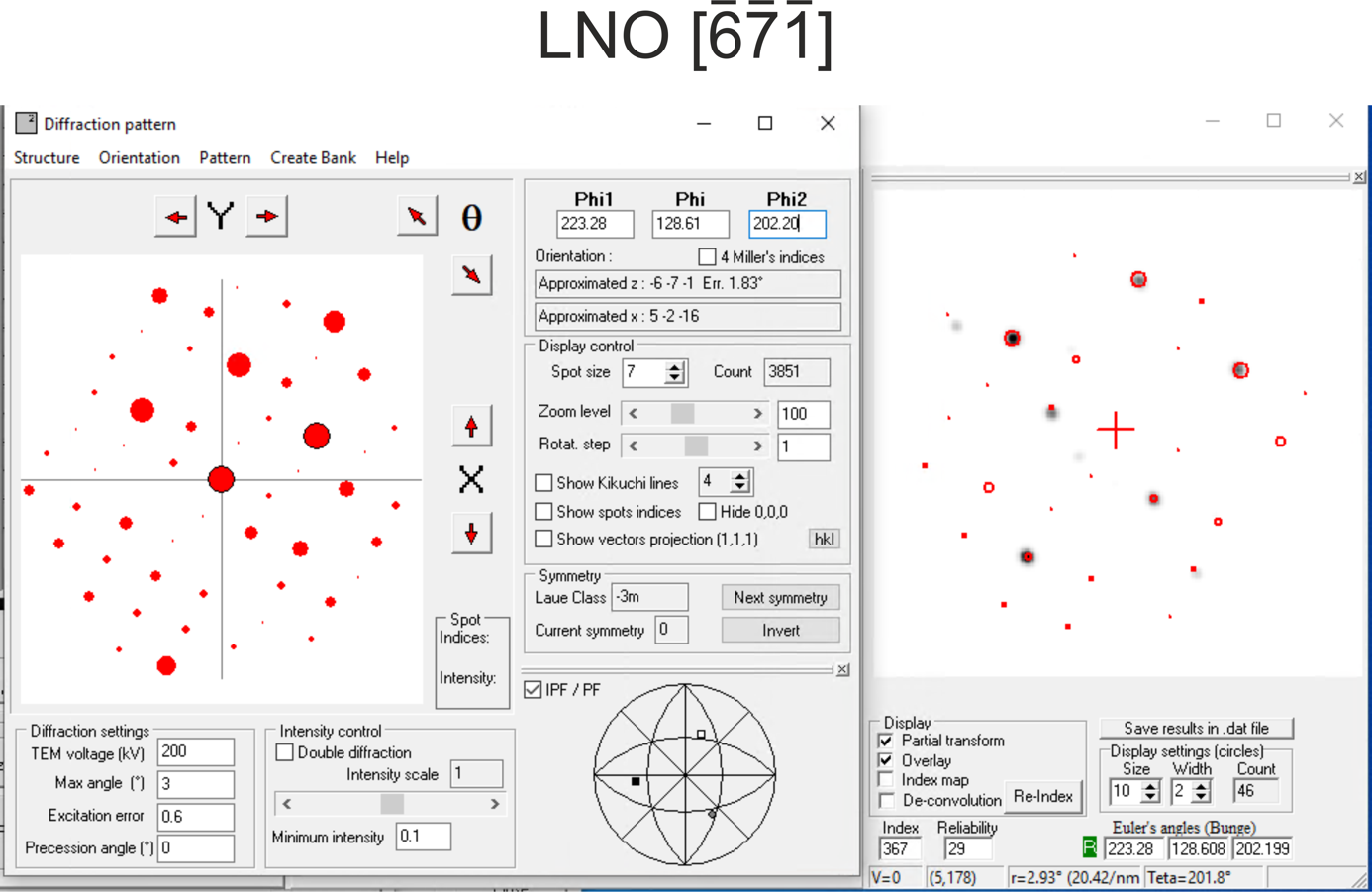


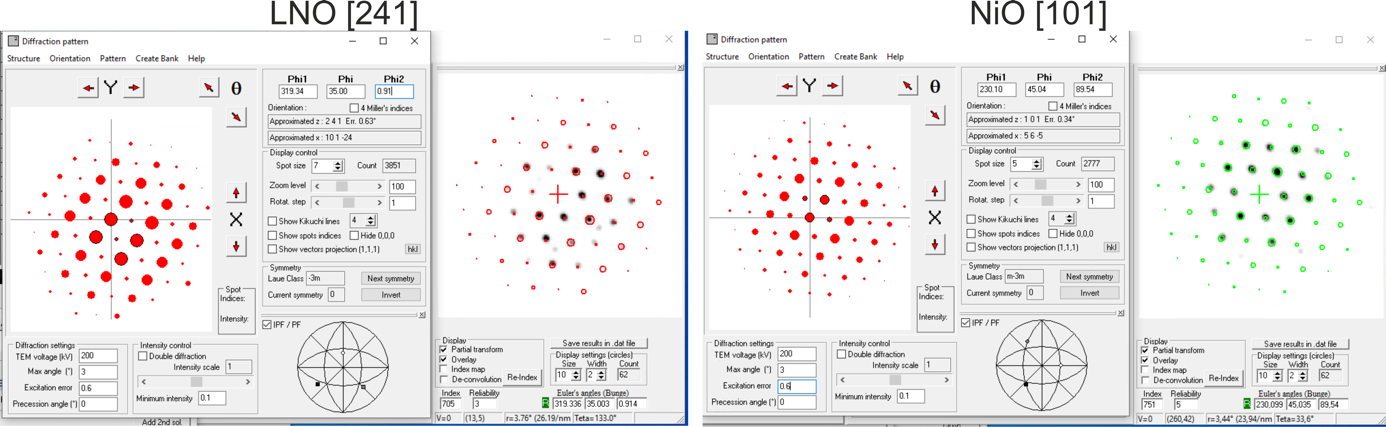


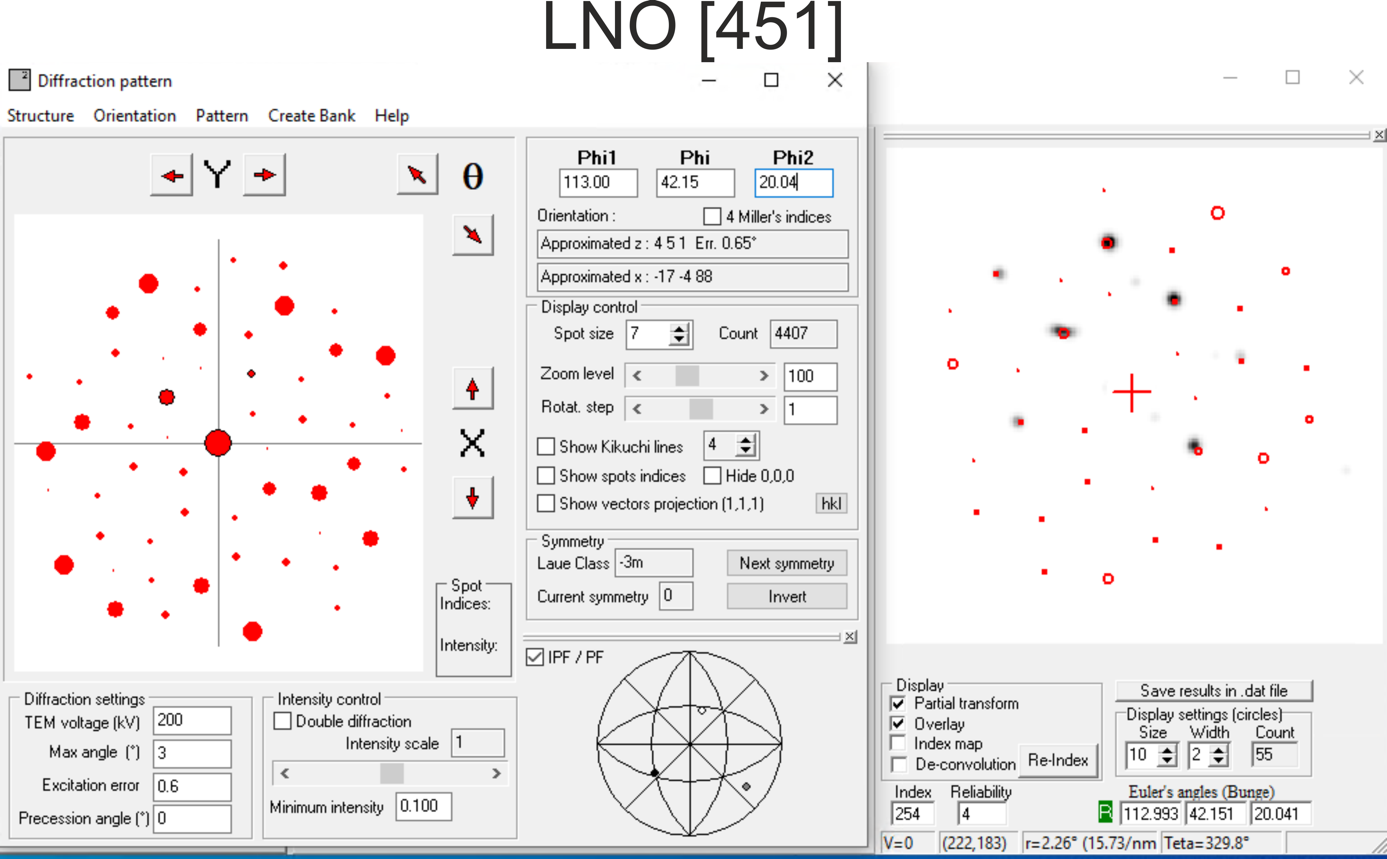


Spinel ($Fd\bar{3}m$) [118]


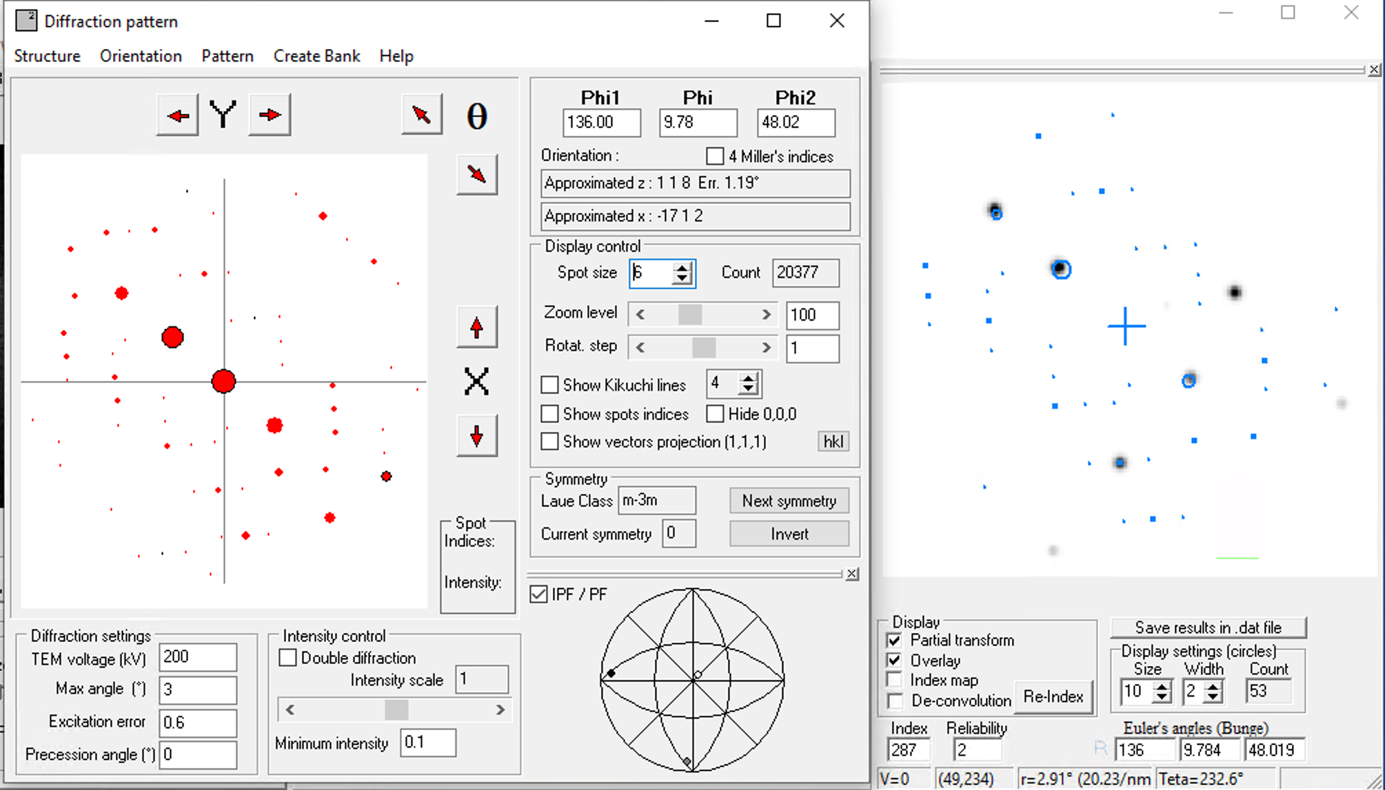


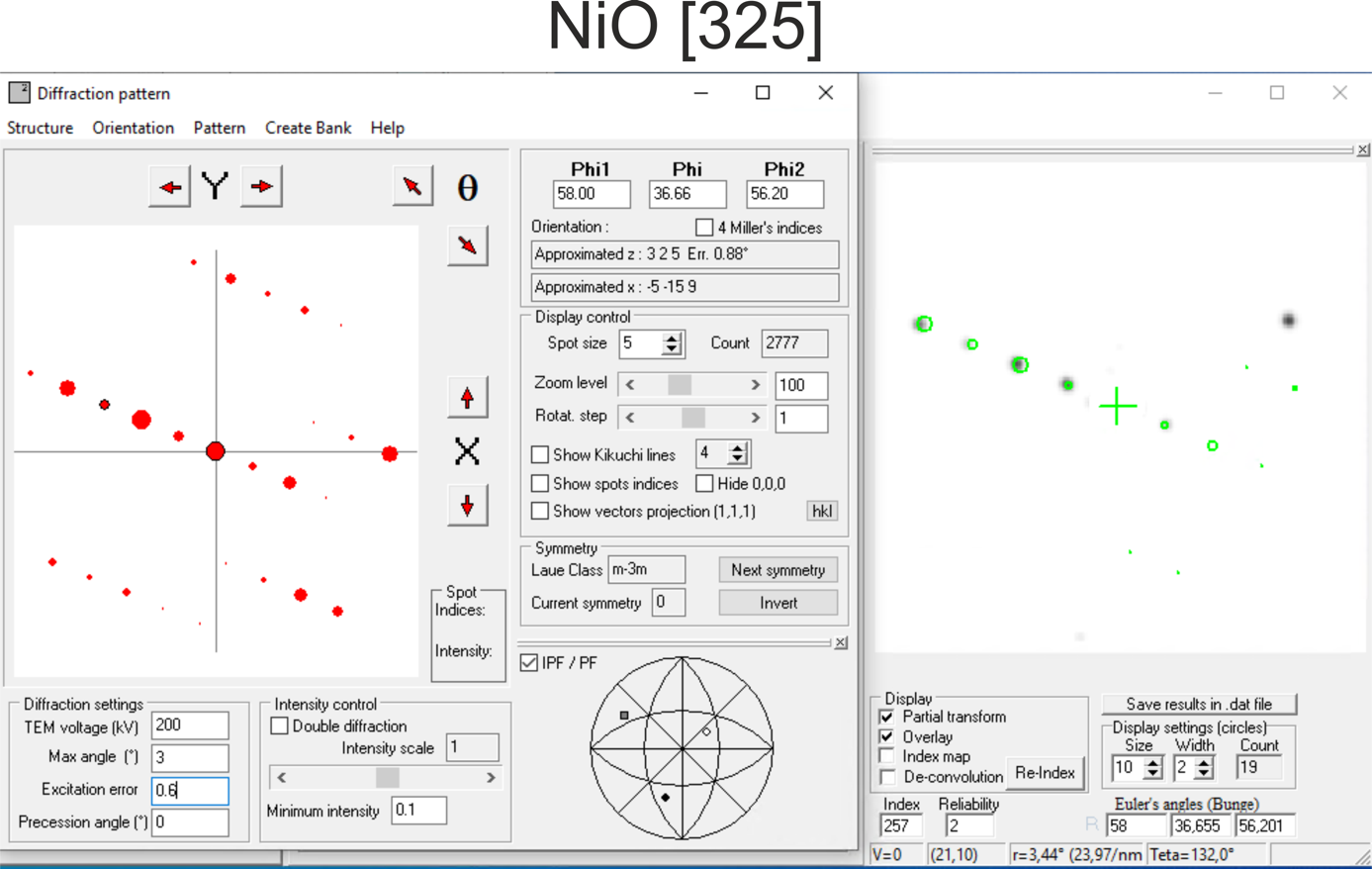


Spinel ($Fd\bar{3}m$) [212]


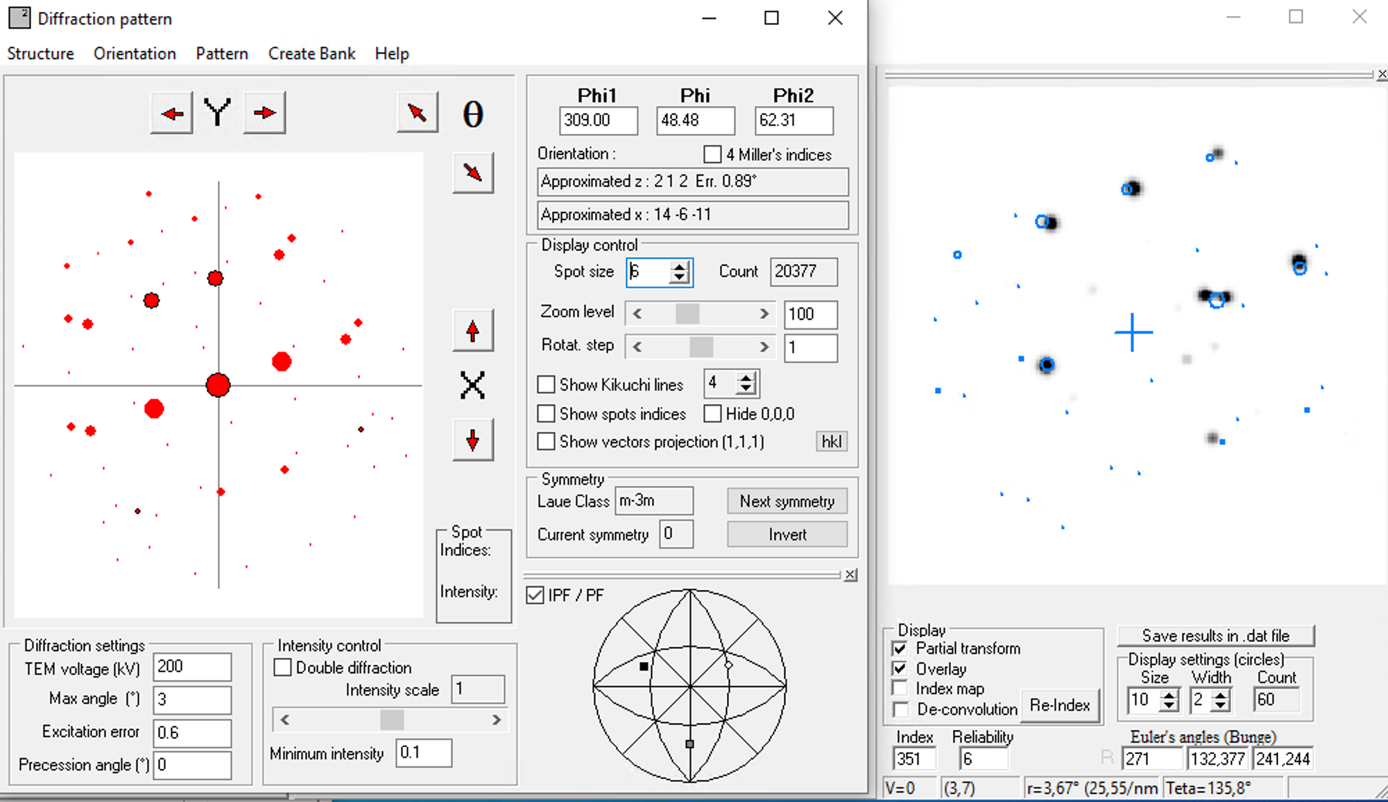


Spinel ($Imma$) [$\bar{1}\bar{1}0$]
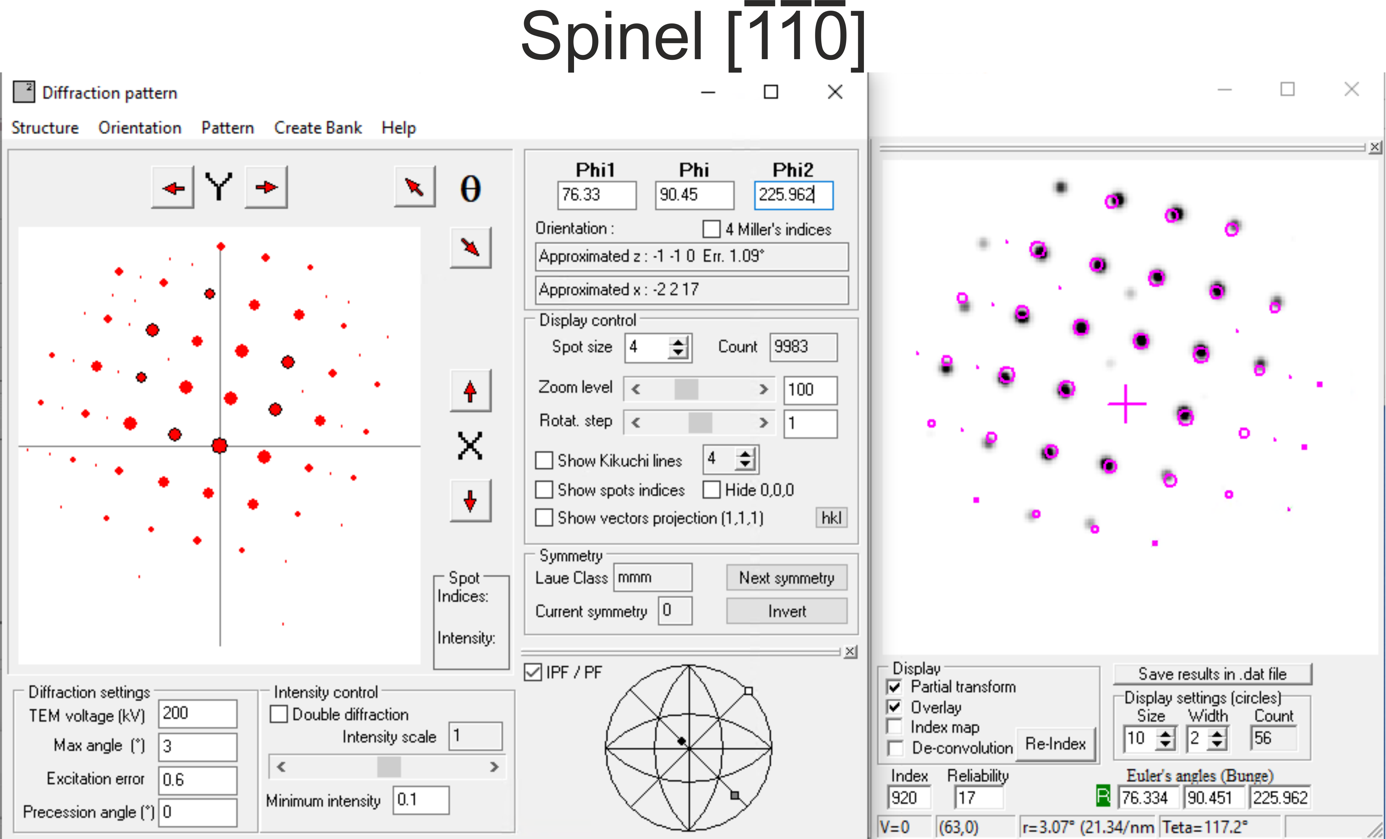


Spinel ($Fd\bar{3}m$) [9 6 23] + NiO [328]


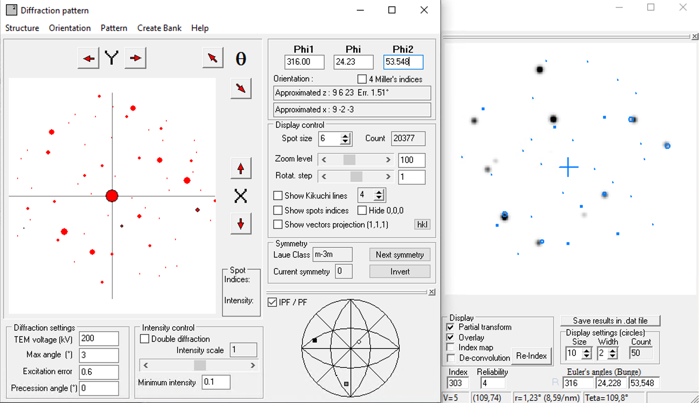

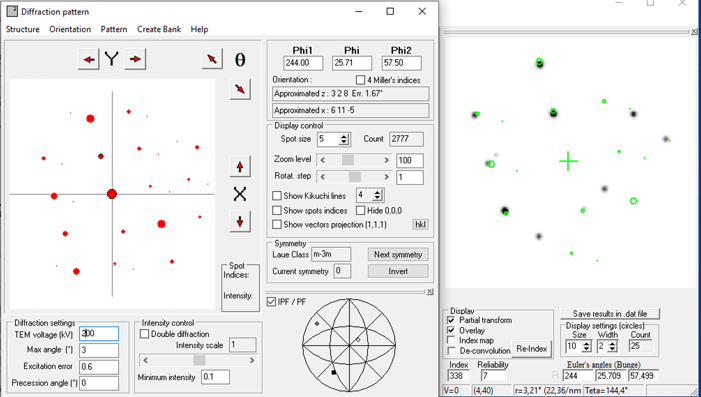


Spinel ($Fd\bar{3}m$) [$\bar{3}\bar{3}\bar{4}$]


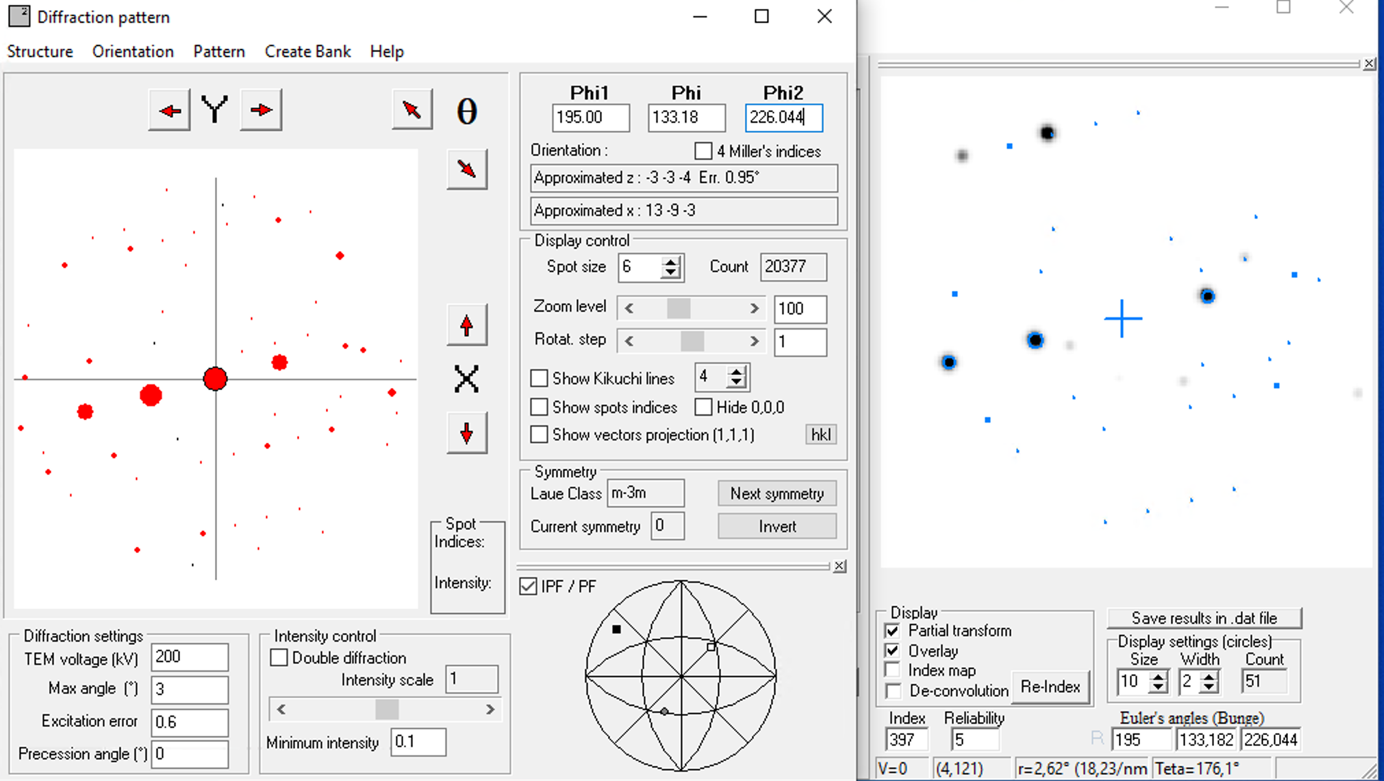


**Figure S4:** Comparison between LiNiO_2_ and NiO. a) An orientation where LNO and NiO are structurally similar and cannot easily be distinguished from an HR-STEM image. b) An orientation where the Li layers in LNO are visible so it is very easy to distinguish from NiO.


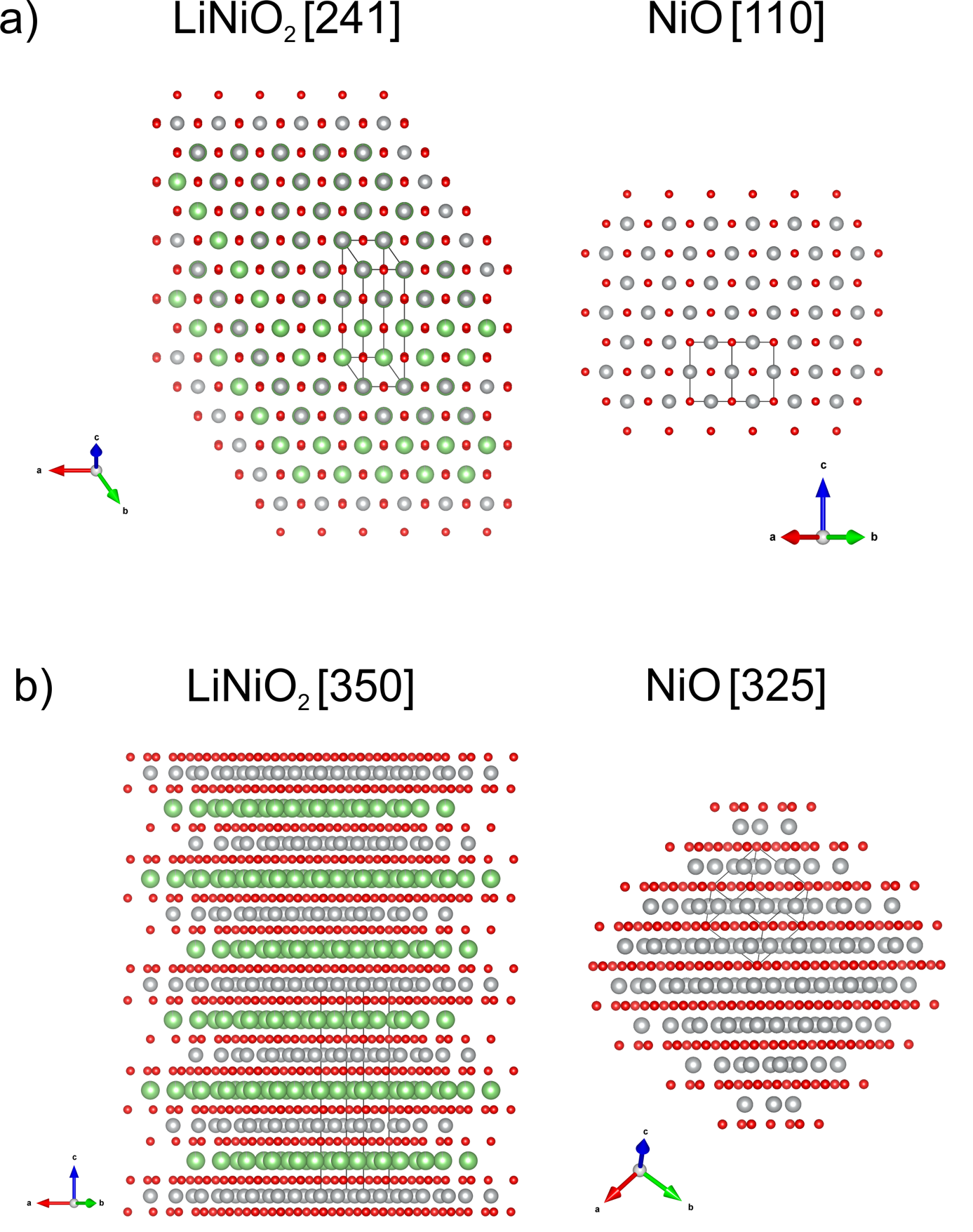


**Figure S5:** HRSTEM image of the heated particle showing a cubic structure. In the inset, the crystal structures of NiO [100] and LiNiO_2_ [841] are overlayed.


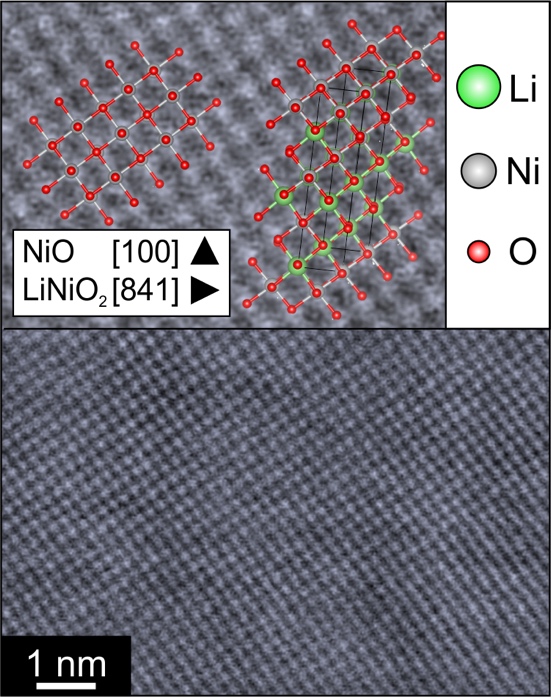


**Figure S6:** Comparison of LNO [241] and NiO [101] orientations with lattice planes (2-10) and (-101) representing the direction of the cracks in the grain, respectively.


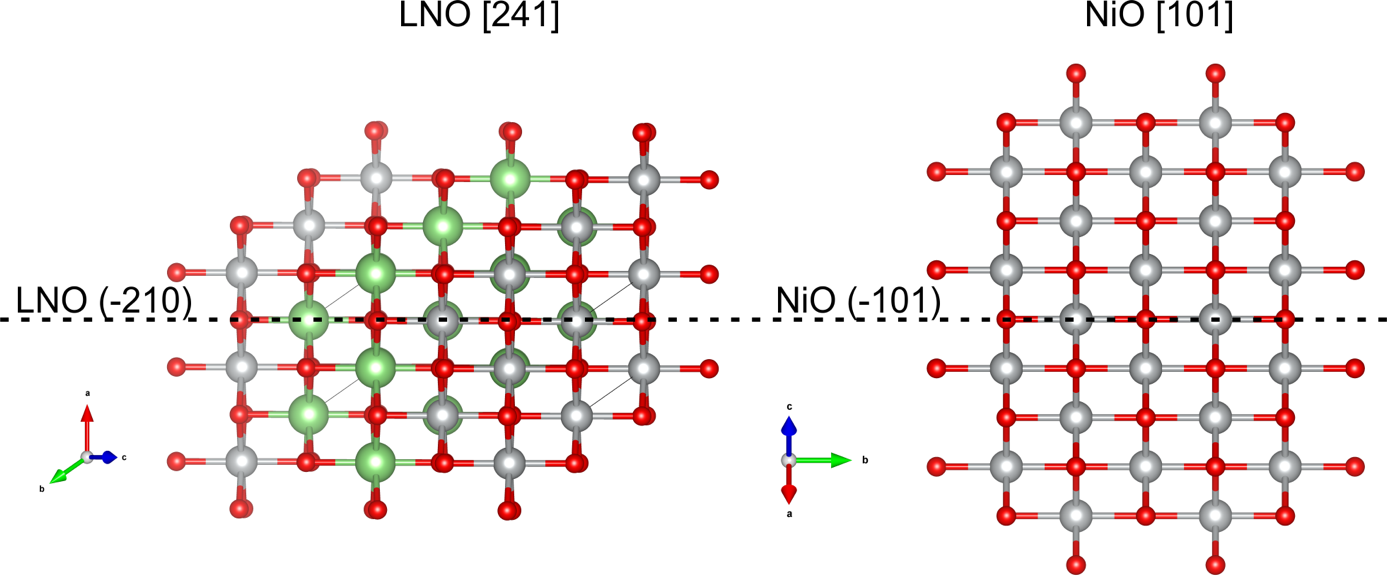


**Figure S7:** a) HAADF HRSTEM image showing lattice planes within the particles on the surface. b) HAADF STEM image showing the particles decorating the grain boundary and the corresponding EDXS map highlighting the Au signal.


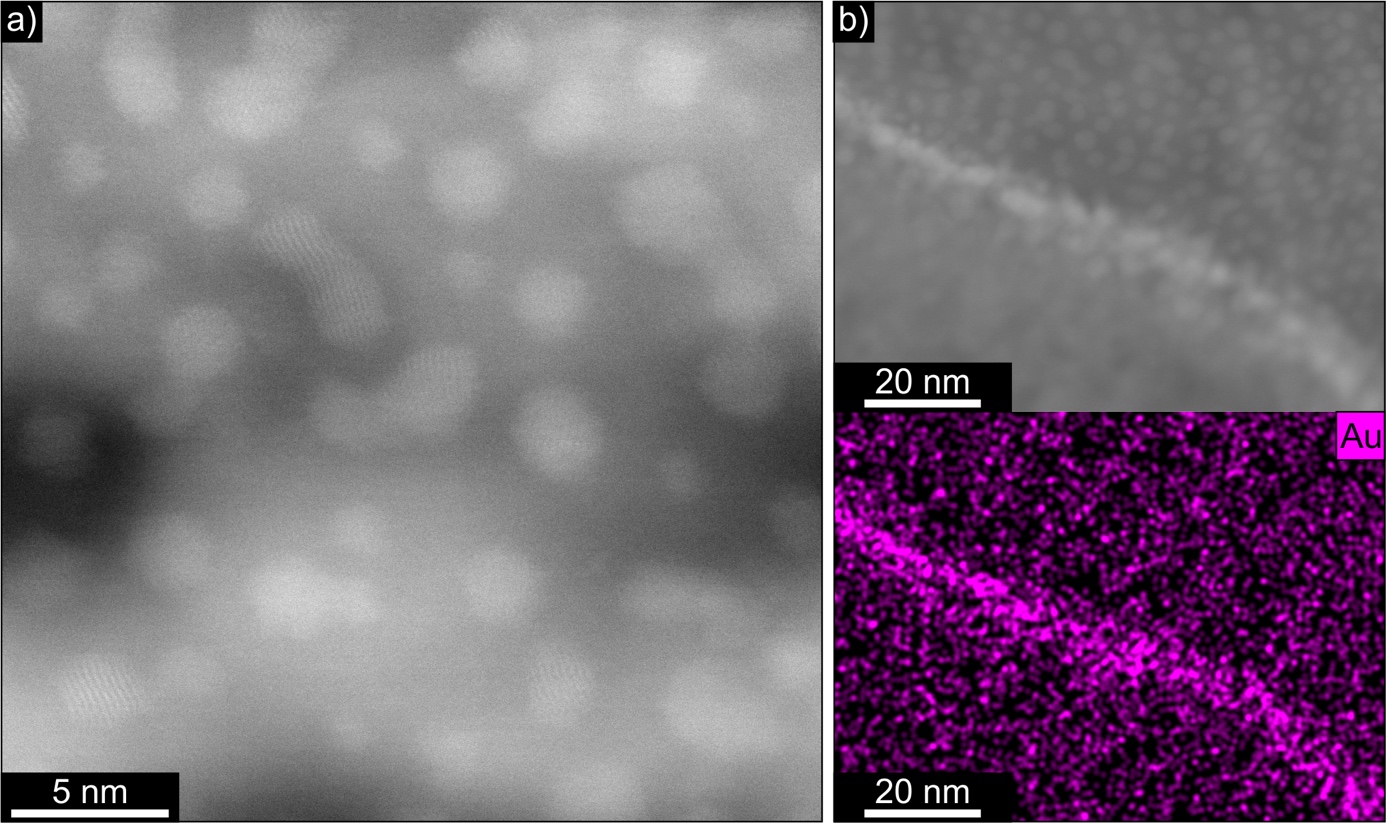

Supplement: Supplementary file 1 — Supporting Information [file SMTD-10-2500357-s002.docx]
